# Inhibitor Development against p7 Channel in Hepatitis C Virus

**DOI:** 10.3390/molecules26051350

**Published:** 2021-03-03

**Authors:** Shukun Wei, Xiaoyou Hu, Lingyu Du, Linlin Zhao, Hongjuan Xue, Chaolun Liu, James J. Chou, Jin Zhong, Yimin Tong, Shuqing Wang, Bo OuYang

**Affiliations:** 1 State Key Laboratory of Molecular Biology, Center for Excellence in Molecular Cell Science, Shanghai Institute of Biochemistry and Cell Biology, Chinese Academy of Sciences, University of Chinese Academy of Sciences, 333 Haike Road, Shanghai 201203, China; weishukun2017@sibcb.ac.cn (S.W.); dulingyu@sibcb.ac.cn (L.D.); lzhao@crystal.harvard.edu (L.Z.); 2 University of Chinese Academy of Sciences, Beijing 100049, China; xyhu@ips.ac.cn (X.H.); jzhong@ips.ac.cn (J.Z.); 3 CAS Key Laboratory of Molecular Virology and Immunology, Institute Pasteur of Shanghai, Chinese Academy of Sciences, Shanghai 200031, China; 4 Department of Biological Chemistry and Molecular Pharmacology, Harvard Medical School, Boston, MA 02115, USA; james_chou@hms.harvard.edu; 5 National Facility for Protein Science in Shanghai, ZhangJiang Lab, Shanghai Advanced Research Institute, Chinese Academy of Sciences, Shanghai 201210, China; xuehongjuan@sari.ac.cn; 6 ShanghaiTech University, Shanghai 201210, China; clliu@ips.ac.cn; 7 School of Pharmacy, Tianjin Medical University, Tianjin 300070, China

**Keywords:** rational design, p7 inhibitors, docking, MD simulation, HCV production

## Abstract

Hepatitis C Virus (HCV) is the key cause of chronic and severe liver diseases. The recent direct-acting antiviral agents have shown the clinical success on HCV-related diseases, but the rapid HCV mutations of the virus highlight the sustaining necessity to develop new drugs. p7, the viroporin protein from HCV, has been sought after as a potential anti-HCV drug target. Several classes of compounds, such as amantadine and rimantadine have been testified for p7 inhibition. However, the efficacies of these compounds are not high. Here, we screened some novel p7 inhibitors with amantadine scaffold for the inhibitor development. The dissociation constant (*K*d) of 42 ARD-series compounds were determined by nuclear magnetic resonance (NMR) titrations. The efficacies of the two best inhibitors, ARD87 and ARD112, were further confirmed using viral production assay. The binding mode analysis and binding stability for the strongest inhibitor were deciphered by molecular dynamics (MD) simulation. These ARD-series compounds together with 49 previously published compounds were further analyzed by molecular docking. Key pharmacophores were identified among the structure-similar compounds. Our studies suggest that different functional groups are highly correlated with the efficacy for inhibiting p7 of HCV, in which hydrophobic interactions are the dominant forces for the inhibition potency. Our findings provide guiding principles for designing higher affinity inhibitors of p7 as potential anti-HCV drug candidates.

## 1. Introduction

Hepatitis C is a type of chronic liver diseases that only occurs in humans and chimpanzees in a slow and unnoticeable manner [1]. Chronic hepatitis C may lead to liver cirrhosis, fibrosis and even hepatocellular carcinoma. It is currently estimated that hepatitis C infection affects approximately 185 million people worldwide which causes up to 500,000 deaths every year [2,3]. For a long-standing period, hepatitis C has been a global health problem due to the lack of an effective vaccine or approved therapies specifically targeting on hepatitis C virus (HCV), the major cause of hepatitis C. HCV is a positively-stranded RNA virus that was first discovered in 1989 [4]. Until now eight main HCV genotypes have been identified through phylogenetic analysis of HCV isolates worldwide [5]. Moreover, each genotype is further subdivided into dozens of subtypes which consist of genetic variations between diverse strains [6]. The viral genetic mutations affect the course of diseases and the responses to antiviral therapy pronouncedly.

Recently, a wealth of direct-acting antiviral agents (DAA) have been designed against hepatitis C virus, primarily targeting three non-structural proteins (NS3/4A protease, NS5A protein and NS5B RNA polymerases) with different mechanisms, respectively [7,8,9,10]. Telaprevir and boceprevir are the protease inhibitors of NS3/4A protease. Daclatasvir can inhibit RNA replication and virion assembly of HCV through blocking NS5A protein. Sofosbuvir is a nucleoside/-tide analogue which can prevent NS5B RNA polymerase from transcribing HCV genomes [11]. Though minor side effects exist, and some patients didn’t respond to these drugs, the current DAA therapies have paved the way to cure the HCV-infected patients. However, the emergence of viral resistance to these drugs is always a particular concern to the society [12,13]. Thus, developing new drugs and targeting a brand-new protein should be continuously under exploration.

p7, a small hydrophobic protein located between the structural and non-structural regions of the HCV polyproteins on the endoplasmic reticulum [14,15], plays an essential role in viral assembly and release [16]. Recent studies have developed a high-throughput profiling platform and found that p7 of HCV has an immune evasion function [17]. p7 can also be pursued as a potential vaccine antigen to induce multifunctional CD4+ and CD8+ T-cells to eliminate hepatocytes that express the targeted HCV antigen [18]. Moreover, p7 oligomerizes to form a 42-kDa hexamer cation channel with a flower-like shape [19,20] and a variety of prototypic small molecules, including amantadine, BIT225, hexamethylene amiloride (HMA) and etc., have been demonstrated to block its activity, which hold p7 high as a potential antiviral target in the HCV life cycle [16,21,22,23,24]. However, variable efficacies have been found for these diverse inhibitors to block p7 channel activity. Amantadine and rimantadine did not display significant benefits against HCV in clinical trials. BIT225 displays a higher potency in the antiviral activity against HCV as a candidate in clinical trials [25], while HMA [23] and the long-alkyl-chain iminosugar derivatives [26] suppress HCV p7 ion channel signals at μM range concentrations. The in vitro studies also showed substantial differences in the p7 inhibition in different HCV genotypes [16].

Due to the essential role of p7 in HCV and its possible use in antiviral therapy, the structure of p7 viroporin has been studied extensively. Initial attempts revealed that p7 forms a mix of hexameric and heptameric complexes in liposomes [27]. NMR-based structures of the HCV p7 monomer have been determined and displayed a hairpin-like conformation [28]. An electron microscopy (EM) study provided the first 3D structure of hexameric p7 channels at a resolution of 16 Å in DHPC (1,2-diheptanoyl-snglycero-3-phosphocholine) micelles [29]. Our previously reported NMR structure showed a hexameric oligomer in DPC (dodecylphosphocholine) micelles at atomic resolution [20], which exhibits a funnel-like shape resembling the EM-based 3D model and fitting within its EM map with high accuracy. Taking advantage of the 3D structure of the p7 hexamer, the drug binding sites of amantadine and rimantadine were further identified by ^15^N-edited nuclear Overhauser enhancement spectroscopy (NOESY) spectra and verified by two-electrode voltage-clamp technique [20]. We also determined the binding of HMA to p7 using NMR combined with molecular dynamics [30]. The results showed that these compounds with different chemical scaffolds bind to discrete hydrophobic pockets with relatively low binding affinity, but causing similar long-range conformational changes near the narrow opening of the cavity [31].

Many efforts have been devoted to improving the drug inhibition against p7. Shiryaev et al. designed and synthesized some cage compound derivatives based on our solution NMR structure of HCV p7 GT5a (PDB: 2M6X) [20], and evaluated their antiviral activity using Bovine Viral Diarrhea Virus (BVDV) as an HCV model virus [32]. A couple of highly active compounds were identified that may be used as a lead compound in further antiviral drug development. Meanwhile, using the NMR hexameric structure (PDB: 2M6X) [20] and the NMR/MD structure of the monomeric form of GT1b [28] as templates, symmetric dimeric adamantane derivatives were designed and synthesized, where two cage molecules were linked by various alkyl-spacers [33]. These dimeric molecules tested in different HCV genotypes were designed for conferring more binding propensities into p7 pocket. Several dimeric compounds showed better potency than that of amantadine in some genotypes [33,34,35]. A recent rational designed compound with an oxindole scaffold displayed strong anti-HCV activity [36]. These studies achieved some improvements on the inhibition of p7; however, the inhibitory effects on the p7 channel are still at relatively low drug efficacies. Therefore, there is still a long way to find new anti-hepatitis drugs.

Here, we also synthesized and screened various novel inhibitors using amantadine and rimantadine as the scaffold, therefore named as “Amantadine or Rimantadine Derivatives” (abbreviated as ARD). The dissociation constant (*K*d) for these ARD-series compounds was determined by NMR-based drug-binding experiments on p7 viroporin protein. The inhibition effects of the strong binders were further confirmed by the antiviral assays. Using molecular dynamics (MD) simulations, we investigated the binding modes and binding stability for the strongest inhibitor, ARD112. We further analyzed the energy data and structural features of drug-protein binding for ARD-series compounds and previously published similar compounds by molecular docking. The in silico-binding affinities identified in the analysis are consistent with our NMR results and previously published data, which shows that the hydrophobic interactions are the main driving force for drug binding. Extra forces provided by several functional groups, including phenyl, halogen and etc., enhance the drug-protein interactions. The detailed tips based on the types of interaction forces between ligands and p7 from our study could provide clues for the future anti-p7 drug design.

## 2. Results

### 2.1. Compound Design

Amantadine is an approved antiviral medicine that was used to block the M2 channel in influenza A virus and treat or prevent influenza infections, which makes it a good lead compound for the drug design. However, amantadine only showed weak inhibition activities on HCV p7, so based on the structural knowledge on the amantadine or rimantadine binding to p7, we designed a series of amantadine or rimantadine derivatives (ARD-series) (Tables S1 and S2) to improve the binding affinity. The approaches for the inhibition enhancement mainly focused on introducing more favorable hydrophilic contacts to the channel lumen or increasing the lipophilic interactions within the hydrophobic pockets.

### 2.2. NMR Titrations of ARD-Series Compounds

**Figure 1:**
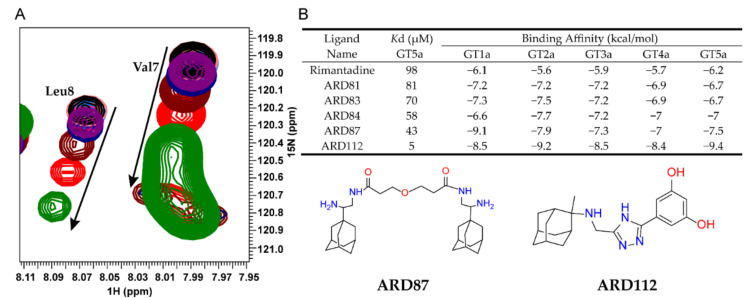
NMR-based screening for ARD-series compounds. (**A**) Examples of residue-specific chemical shift perturbations at various compound concentrations: 0 (black), 0.1 (midpink), 0.2 (skyblue), 0.4 (pink), 0.8 (purple), 1 (navy), 2 (maroon), 4 (red) and 8 mM (green). The peaks are from 2D ^1^H-^15^N TROSY-HSQC spectra recorded at 600 MHz (^1^H frequency) with a 0.1 mM U-^15^N-p7 sample reconstituted in 20 mM DPC. Chemical shift changes of residues Val7 or Leu8 were used as the indicators for calculation of *K*d values. (**B**) *K*d values were calculated by fitting the titration data to a standard equilibrium binding equation. The *K*d values obtained from NMR titrations and the binding affinities of the top 5 compounds to 5 genotypes are shown. The chemical structures of ARD87 and ARD112 are depicted at the bottom panel.

To characterize the interactions between ARD-series compounds and p7, we titrated p7 (5a) protein with the successfully synthesized ARD-series compounds and monitored compound-induced chemical shift changes using 2D TROSY-HSQC (transverse relaxation optimized spectroscopy and heteronuclear single-quantum coherence). The p7 (5a) protein was prepared as previously described [20]. Rimantadine is a known inhibitor of p7 activity and was used as a benchmark for comparison. The NMR titrations showed residue-specific and concentration-dependent chemical shift changes caused by ARD-series compounds similar to those induced by rimantadine (Figure 1A). The inhibitory effect can be revealed through the binding interaction with the target protein, thus the dissociation constant (*K*d) calculated from chemical shift changes were used to compare the inhibitory effect for screened compounds (Figure 1B and Table S1). The apparent *K*d values of Val7 or Leu8 were used as indicators since previous studies have demonstrated that the structural rearrangements at the bottleneck area of p7 channel are very crucial for ion passage. We noted that due to the partition of the hydrophobic compounds in detergent micelles, the effective compound concentrations are difficult to estimate, and the apparent *K*d values are all relatively high.

The binding affinities for screened compounds were listed in Table S1. Compared to rimantadine, many ARD-series compounds showed weaker affinity. Only a few compounds were more potent than rimantadine, including ARD81, ARD83, ARD84, ARD87, and ARD112. We found most of the dimeric adamantane ARD-compounds (ARD81, ARD83, ARD84, ARD87) are more potent or equipotent to rimantadine which is consistent with the previous study [33]. ARD112, the strongest binder among all of the screened compounds, which contains a hydrophobic cage linked with a phenyl group and a triazole group, is ~10-fold stronger than the best dimeric compound, ARD87.

### 2.3. The Antiviral Activity and Cytotoxicity of Synthetic ARD87 and ARD112

**Figure 2:**
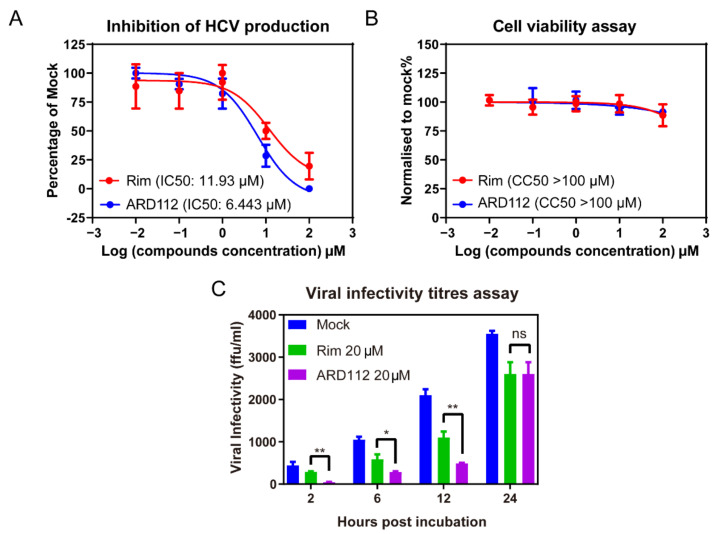
The p7 inhibitor ARD112 can inhibit HCV production. (**A**) The inhibition of HCV production was determined. Huh7.5.1 cells infected by JFH1 at an MOI of 2 for 1 day were treated with a serial dilution of Rim or ADR112. 12 h later, the viral production was detected by the titration assay. (**B**) After incubation with each compound, the cell viability and cytotoxicity were detected by CellTiter-Glo^®^ Luminescent Cell Viability Assay reagent (Promega GS7570, Madison, WI, USA). (**C**) Time course of HCV production with the treatment of ARD112 and Rim at concentration of 20 μM. Data were presented as the percentage of mock treatment control. The error bars were calculated from three individual measurements (* refers to *p* < 0.1, ** refers to *p* < 0.05, ns: no significance, ARD112 compared to Rim). Two independent experiments were repeated showing similar results.

To further verify the inhibition effect of ARD87 and ARD112 against HCV, we tested the inhibition of HCV production with incubation of ARD87 and ARD112 using rimantadine (Rim) as a control, which was reported to possess moderate anti-HCV activity but no significant cytotoxicity against hepatoma cell lines [35]. HCV infected Huh7.5.1 cells treated with a serial dilution of each compound for 12 h, respectively. Then the viral infectivity titers were detected by the titration assay. As shown in Figure 2A and Figure S1A, the IC50 of ARD87 and ARD112 against HCV was 5.14 μM and 6.42 μM, respectively, about two-fold lower than that of Rim. As shown in Figure 2B, the cytotoxicity of the ARD112 is comparable to Rim, while the cytotoxicity of the ARD87 is significantly higher than Rim (Figure S1B). The viral production kinetics was calculated by collecting the supernatant at 2-h, 6-h, 12-h and 24-h post-incubation and the infectivity titers were detected by the titration assay. The results demonstrated that incubation with 20 μM ARD87, ARD112 or Rim could suppress the viral production comparing to the mock control (Figure 2C and Figure S1C).

### 2.4. Molecular Dynamics Simulation of p7-Ligand Complex

**Figure 3:**
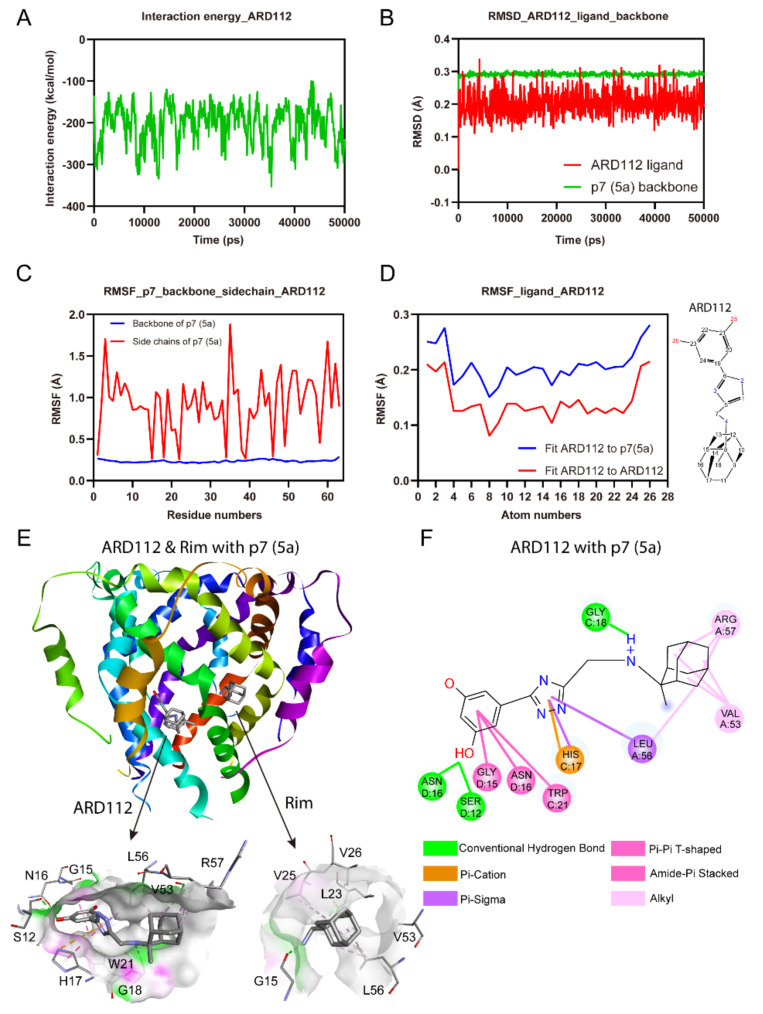
The stability of ARD112 with p7 (5a) in molecular dynamics simulation. RMSD or RMSF has three levels to show stableness, below 5 Å (low stability), below 3 Å (medium stability), below 1 Å (high stability). (**A**) The interaction energies of ARD112 and p7 (5a) complex. The channel structure with the inhibitor binding were maintained over the entire trajectory. (**B**) The RMSD values of the protein and inhibitor heavy atoms from their starting positions of the p7-inhibitor complex. (**C**) The RMSF values of the complex backbones and sidechains over the entire trajectory. (**D**) The RMSF values of ARD112 atoms are less than 1 Å, which is perfectly acceptable to protein and ligand itself from the start to the end. (**E**) Comparison of 3D-binding plots of rimantadine and ARD112 with p7 (5a). The binding sites were shown in the zoomed views at the bottom. (**F**) Detailed bonding between ARD112 and p7 (5a), interaction forces including hydrogen bonding, Pi-Pi T-shaped, Pi-cation, amide-Pi stacked, Pi-sigma and alkyl forces. Note: Shadow refers to the solvent accessibility area, A-F refers to the chain ID, digital number refers to residue number in the light of residue name.

To independently investigate the binding site and complex stability for ARD112 and to decipher the favorable interactions, we performed MD simulation with HCV p7 channel (PDB Code: 2M6X) [20]. The p7-ligand complex conformations obtained by molecular docking were used as starting models for simulations. For simplicity, the p7 viroporin was inserted into a membrane of a single-component 1-palmitoyl-2-oleoyl-sn-glycero-3-phosphocholine (POPC) lipid bilayer built by Desmond [37]. The whole processes of MD simulations were under the condition of an ionic strength of 0.15 M NaCl buffer. Finally, the production process of channel-drug/lipid system was conducted under the OPLS2005 force field [38] to capture the dynamic trajectories for 50 ns after the heating and equilibration processes. MD simulation showed that the interaction energies of ARD112 and p7 (5a) complex are low (Figure 3A) and the overall six-fold symmetry is retained during the simulation, while the starting structure undergoes some local distortions with the tethering of protein backbone.

The dynamic stability of the channel-drug complex was elucidated by calculating the Root-Mean-Square Deviation (RMSD) values for the protein and inhibitor atoms, respectively. It was calculated for all the frames in 50 ns trajectory. The average RMSD values of ARD112 ligand atoms and the p7 backbone is around 0.2 and 0.3 Å, respectively, indicating that the complex is stable (Figure 3B). The Root Mean Square Fluctuation (RMSF) is useful for characterizing local changes along the p7 backbone and sidechains. The analysis of RMSF of complex between ARD112 and p7 (5a) showed that sidechains for all the residues display fluctuations between 0.2–2.0 Å and the backbones remain steady at lower values over the entire trajectory (Figure 3C). The Ligand Root Mean Square Fluctuation (L-RMSF) was introduced to characterize changes in the ligand atom positions, which gives insights on how ARD112 fragments interact with p7 and their entropic roles in the binding event. The L-RMSF values retained less than 0.3 Å on each atom through the whole process, which reflects the small internal atom fluctuations of ARD112 (Figure 3D). Both RMSD and RMSF analyses confirmed the structural stability of the whole system; thus, MD simulation results are suitable for further analysis. In contrast, ARD87 showed relatively higher and more fluctuated interaction energies, RMSD and RMSF values than those of ARD112 (Figure S2), which is consistent with its relatively weaker binding affinity.

For complex of ARD112 with p7 (5a), as shown in Figure 3E, no obvious clashes are observed in the interactions for the stabilization. The hydroxyl groups on aromatic ring of ARD112 average points to the channel lumen. This compound is accommodated into the space between helices, binding to residues consisting of S12, G15, N16, H17, G18, W21, V53, L56 and R57 (Figure 3E). The hydrophobic residues build strong hydrophobic connections with different parts of ARD112. Specifically, the aromatic ring of ARD112 display Pi-Pi T-shaped interaction with W21 and amide-Pi stacked interactions with G15 and N16 residues from contiguous chains. Moreover, the Pi-cation force with H17 and Pi-sigma force with L56 for the triazole of ARD112 contribute significantly to the complex stability (Figure 3F). Two hydrogen bonds (S12, 2.3 Å; N16, 2.9 Å) were observed with one of the hydroxyl groups in ARD112.

### 2.5. HCV p7 Channel Modeling

**Figure 4:**
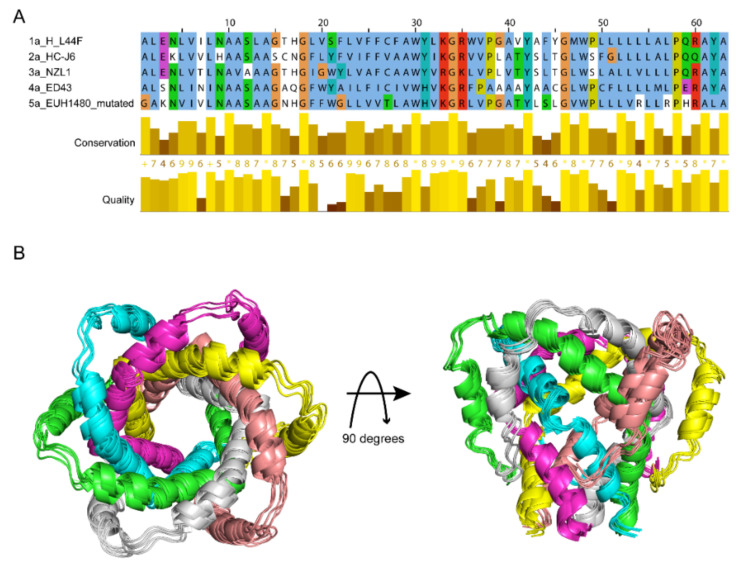
Comparison of p7 sequences and structures from different HCV genotypes. (**A**) Sequence alignment of p7 from genotypes 1a, 2a, 3a, 4a and 5a, showing the conserved residues among the 5 genotypes. (**B**) The overlayed structure models of p7 from genotypes 1a, 2a, 3a, 4a, 5a, in which six chains are colored as purple, yellow, midpink, grey, green and blue. The structure models of p7 (1a) to (4a) were built using PDB ID 2M6X [20] of p7 (5a) by software Discovery Studio 4.0 [39].

For stating the discrepancy among genotypes, models of p7 (1a, 2a, 3a, 4a) were generated from the template structure of p7 (5a) [20] using virtual mutations. The representative p7 sequences for the targeted HCV genotypes Gt1a, Gt2a, Gt3a and Gt4a were obtained from the UniProtKB database (Gt1a: P27958, Gt2a: P26660, Gt3a: Q81258, Gt4a: O39929, respectively). All the protein sequences used in the present study are aligned in Figure 4A. The identity level of genotypes 1a to 4a are above 40% that the modelling accuracy is qualified for further study based on template 5a. Moreover, genetic variations between the sequences are presented in the binding site regions, confirming the representativity of the selected targets. The final structure models for different genotypes showed very similar structures (Figure 4B).

### 2.6. Molecular Docking of Potential p7 Inhibitors Categorized by the Structure

Previously, the biological evaluation of 40 designed compounds (Table S3) [32] were conducted on HCV surrogate BVDV strain VK-1, and 9 symmetric compounds (Table S3) [33] were conducted through 5 genotypes. To obtain more hints for drug design, we performed the molecular docking to 91 compounds, including 42 ARD-series compounds and 49 previously reported compounds through 5 genotypes: Gt1a, Gt2a, Gt3a, Gt4a and Gt5a and predicted their binding modes and binding energies to evaluate the binding potency of these drug candidates (Tables S1 and S3).

Taking into account their basic nature, 91 compounds used in protonated and minimized forms were docked into the representative binding sites of five channel variants using the Autodock Vina 1.1.2 software [40]. Over the entire library through 5 genotypes, binding affinities were calculated from the built-in algorithm which are distributed in a range from −9.4 to −4.8 kcal/mol (mean value −6.65 kcal/mol). The average binding energy of all the compounds over given genotypes varies from −6.88 to −6.41 kcal/mol (mean value −6.65 kcal/mol) (Figure S3A–E). The major binding types for each ligand are hydrophobic interactions and hydrogen bonds (Figure S3F).

For 91 compounds, the binding energies identified by docking showed the similar trend as NMR titration data or BVDV inhibition, which could be reasonably used as an indicator to evaluate the inhibition effects of p7 channel. For example, the three inhibitory compounds 61, ARD87 and ARD112 showed strong binding affinities (Tables S1 and S3) gained from AutoDock Vina [40] and strong inhibition as indicated by previous BVDV inhibition data [32] or our HCV antiviral assays. Thus, the binding energies are used in the following analyses.

### 2.7. Interaction Forces Contribute to the Binding

**Figure 5:**
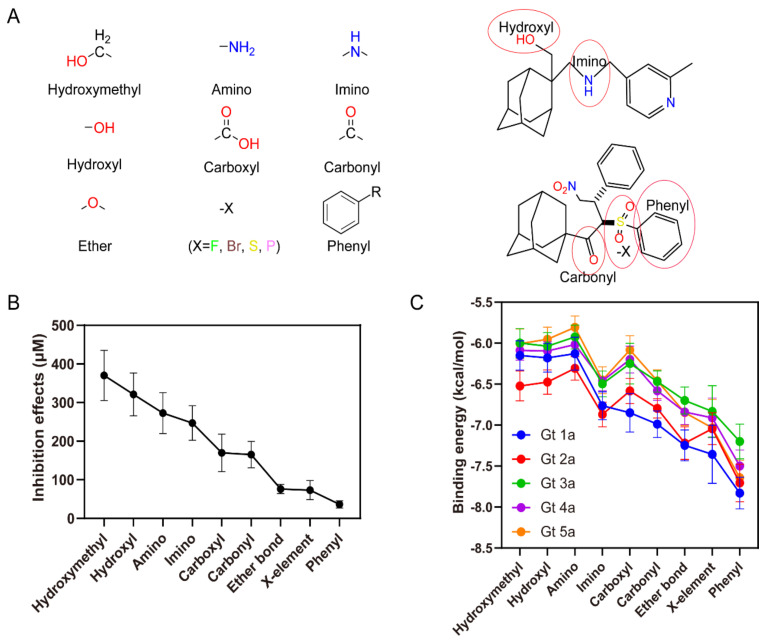
Contributions of different functional groups to inhibition effects and binding energies. (**A**) Left: nine major functional groups presented in the compounds. Right: two examples for the functional groups labeled with red ellipses in the compounds. (**B**) Inhibition or *K*d values categorized by functional groups arranged in the order of effectiveness. These analyses were conducted from experiment data using the mean value of inhibition or *K*d values, standard error of mean value, and number of values for each functional group using the statistics description. (**C**) Binding energies for compounds to p7 from 5 genotypes, categorized by functional groups in the order of effectiveness. The analyses were conducted by molecular docking using the structure models built for p7 Gt1a to Gt5a. Note: These data are derived from the ligands of Table S1 (ligands from ARD1 to ARD112) and Table S3 (ligands from 3 to 79).

To analyze the quantitative structure-activity relationship (QSAR), functional groups from the compounds in this study were classified into 9 major parts, which are hydroxymethyl, amino, imino, hydroxyl, carboxyl, carbonyl, ether bond, halogen and phenyl groups (Figure 5A). From the previous IC50 data [32] and newly calculated *K*d values of ARD-series compounds (Table S1), inhibitory effects of every molecule with diverse functional groups can be analyzed based on the structure-activity relationship. By introducing different functional groups, the inhibition effects mean values range from 36.3 μM to 370.2 μM that the compound inhibition activity increases in the order hydroxymethyl < amino < imino < hydroxyl < carboxyl < carbonyl < ether bond < halogen < phenyl groups (Figure 5B). ARD112 added a phenyl group together with a triazole group, so achieved the strongest inhibition, while adding the hydroxymethyl group had the worst effect on increasing the inhibition inferred from the experiments.

The binding energy analysis shows that the compound binding energies increase in a similar order for different functional groups, except that the amino group has a relatively higher binding energy than the hydroxyl group, which doesn’t follow the same trend as the evaluation based on inhibition effects (Figure 5C). By comparing the binding energies of the compounds (Tables S1 and S3), it was also found that the phenyl functional group is more favorable than other functional groups for the 5 genotypes, which is consistent with the analysis from the inhibition data.

### 2.8. Residue-Specific Contributions to the Binding Affinities

**Figure 6:**
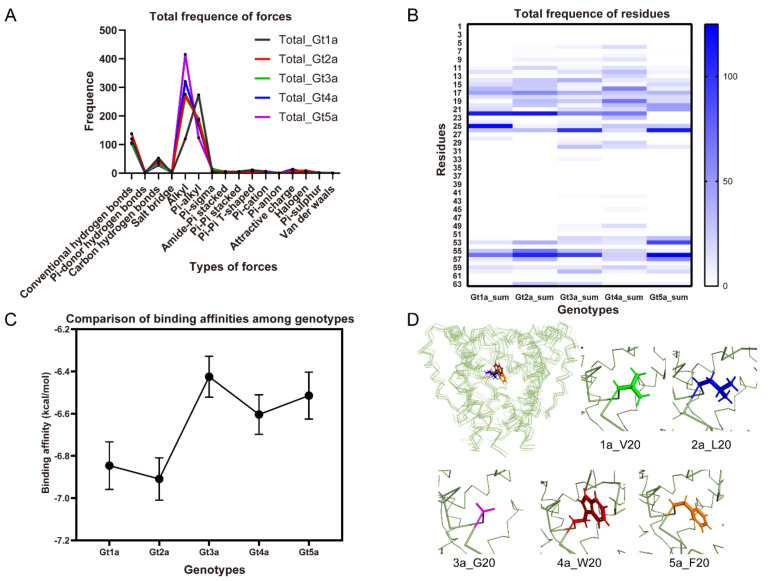
Contributions of p7 residues and interaction forces in the binding affinities. (**A**) Frequence of diverse forces involved in binding between all the compounds and p7 through 5 genotypes, indicating that alkyl and Pi-alkyl hydrophobic forces are the major forces. (**B**) Frequence of interaction forces between all the compounds and p7 through 5 genotypes deciphered the key residues involved in binding with all the ligands. (**C**) Binding affinities calculated from docking were arranged in the mean value plot among each genotype. Black dots refer to the mean value of binding affinities from all the ligands using docking, bars refer to the standard error of mean value. (**D**) Residue 20 is shown as side-chain sticks. Residue 20 is valine (green), leucine (blue), glycine (purple), tryptophan (red), phenylalanine (orange) for Gts 1a, 2a, 3a, 4a, and 5a, respectively. Note: Gt is an acronym for genotypes. These data are derived from all the ligands of Tables S1 and S3.

The docked poses of the compounds were further analyzed to identify the binding region in p7 channels. Statistically, alkyl and Pi-alkyl forces are the two major forces involved in binding interactions with the high frequency through 5 genotypes (Figure 6A). Conventional and carbon hydrogen-bonds also contribute significantly to the inhibition effects. The molecular docking analysis highlight the residues 22, 26 and 56 are highly conserved hydrophobic interactions among all complexes (Figure 6B), which contributes the drug binding to p7 hydrophobic pockets. Thus, hydrophobic interactions play a key role in binding processes. We note that most of the binding energies on genotype 3 are relatively higher than other genotypes (Figure 6C), indicating the weak inhibition activities on genotype 3, which is consistent with the previously reported results that amantadine could not inhibit genotype 3 [41]. The docking is further used to assist the evaluation of genotype-dependent sensitivity of p7 to multiple inhibitors. The largest difference between the five genotypes studied here is residue 20, which is a glycine in Gt3a but valine, leucine, tryptophan, phenylalanine in Gts 1a, 2a, 4a, and 5a, respectively (Figure 6D). Residue 20 is an essential part of the drug pocket and is in direct contact with the adamantane cage. The previously reported L20F mutation in genotype 1b which was identified in clinical trials that confers amantadine resistance also confirmed the importance of residue 20 [42,43]. The missing hydrophobicity of G20 in Gt3a may cause the weak binding ability in this region. Accordingly, it can be concluded that the hydrophobic interactions are the fundamental forces in the structural stability of channel/ligand complex.

## 3. Discussion

The inhibition potential from the high similarity in the viroporin family makes p7 channel an attractive template for antiviral inhibitor design. In the present study, we screened a library of 42 compounds with variable bridge size and various sidechains based on the adamantane scaffold. Our data showed that the synergetic design to combine two parts are useful to increase the binding affinity, which is consistent with the previous studies. Moreover, one lead compound ARD112 was identified as a strong binder of p7 channel, which contains one adamantane cage and one aromatic ring linked with a triazole. Both NMR titrations and MD simulations indicated that ARD112 is a more potent inhibitor than the dimeric adamantane compounds, which could be further optimized for the development of HCV treatment. The subsequent evaluation of ARD112 and a dimeric ARD87 by antiviral assay showed ARD112 has a similar inhibition effect on the HCV production as ARD87, but ARD87 shows a higher cytotoxicity in the hepatoma cell line, which means ARD112 is a better lead compound for the development of HCV treatment. MD simulation-based studies also showed that the aromatic rings are important to improve the binding affinities to p7 channel, which provides a direction to further optimize the compounds in the further drug design. Interestingly, a recently published paper from Shaw et al. also reported a stronger inhibitor-JK3/32, which contained aromatic groups and exerted irreversible blockade on p7 channel [36].

To gain deeper insights into the inhibition effects, detailed molecular docking studies have been also performed to delineate the ligand-protein interactions at molecular levels using Autodock Vina programs [40]. Using experimentally screened compounds, structure activity relationship and the interaction of the compounds with the help of docking studies were established. Interestingly, the binding affinities of the compounds revealed some similar trends through 5 genotypes 1a, 2a, 3a, 4a, and 5a, which indicates the trend of inhibition effects are the same as the binding affinities from the molecular docking. Experimental data and docking data are highly compatible with each other, therefore, the comparison between 5 genotypes were further analyzed based on the docking results. We first evaluated the functional groups to assess which functional groups have beneficial interactions with the target. The interaction forces involved in the binding are hydrogen bonds, electrostatic, hydrophobic, halogen and miscellaneous bonds, in which hydrophobic forces contribute the most for binding among the complexes. The interaction analysis highlights higher structural stability and reduced flexibility in presence of hydrophobic groups, while hydrogen bonds also play an important role among all the ligands (Figure S3F).

We next analyzed residues in the active site of p7 within five genotypes. Such exploration is performed to interpret the model and understand how exactly it correlates activity to specific structural features. No significant differences were detected in the structure models or sequence alignment between the five genotypes (Figure 3). However, some differences in the binding energy were observed that genotype 3a generally displays the highest binding energies for all the compounds. The residue differences in the binding sites of these genotypes are postulated to be the cause of this less affinity of ligands that residue 20 may play an essential role in the drug inhibition activity. The inhibition effects of compounds are not universal among the genotypes, as the interaction forces are observed with given residues in some genotypes but others not, which means the binding and inhibitory response are specific to each HCV genotype. As a result of this, delivering novel inhibitors on specific HCV strains is of foremost importance.

Together, this study illustrates that NMR spectroscopy assisted by molecular docking can provide the dynamic nature of the protein-inhibitor complex which will spark further conversation in the drug-design community to pave the way towards more operable and validating outputs. With growing resistance to the current antiviral agents on HCV [12,13], in addition, the number of treatments available for certain HCV genotypes is limited, keeping paces with resistance through designing new drugs is still enticing. For better and fast design of novel drugs against HCV, a series of tips that is from the results of current NMR titration, MD simulations and published biological evaluation on increasing inhibitory effect may provide useful insights for designing new drugs.

## 4. Materials and Methods

### 4.1. Materials Protein Expression and Purification

The p7 (5a) protein was prepared as previously described [20]. In short, p7 was transformed in *E. Coli* BL21 (DE3) cells fused with His9-trpLE in the pMM-LR6 vector and grown in M9 minimal media. The p7 peptide was expressed in the inclusion bodies and extracted and solubilized in Buffer A (6 M guanidine HCl, 200 mM NaCl, 1% Triton X-100, 50 mM Tris-HCl pH 8.0). The His9-trpLE-p7 fusion protein was first purified by Ni^2+^ affinity chromatography in Buffer A at room temperature and eluted from the Ni^2+^ column in the same buffer with 400 mM imidazole. The eluted protein was then cleaved at the methionine position by cyanogen bromide (CNBr) in 80% formic acid. The p7 and fusion protein were further separated on a Proto-18C column by reverse-phase chromatography. NMR samples were prepared by dissolving 1–2 mg of lyophilized p7 peptide in 6 M guanidine-HCl and 20 mg dodecylphosphocholine (DPC) and refolded by dialysis against the NMR buffer (25 mM MES, pH 6.5).

### 4.2. NMRT Titration for p7 (5a) Oligomer

ARD-series compounds were synthesized by Sundia MediTech Company (Sundia MediTech Co., Ltd., Shanghai, China). A total of 80 ARD-series compounds were synthesized successfully, the results of 42 compounds were reported in this paper, while 38 of them were excluded due to their low solubility. All NMR based small-molecule-titration experiments were recorded at 30 °C using Bruker 600 MHz spectrometer (Bruker Corporation, Karlsruhe, Germany) equipped with a cryogenic probe. All the compounds were dissolved in dimethyl sulfoxide (DMSO) to make stock solutions with final concentrations ranging from 100 mM to 500 mM based on the solubility. The compounds were separately added to a 0.1 mM (^1^H, ^15^N)-labelled p7 (5a) sample reconstituted in 20 mM DPC micelles. Two-dimensional ^1^H-^15^N TROSY-HSQC spectra were recorded at each of the inhibitor concentrations including 0, 0.1, 0.2, 0.4, 0.8, 2, 4 and 8 mM (see Figure 1A). NMR spectra were processed using NMRPipe (Ad Bax Group at the NIH, Bethesda, USA) [44]. The chemical shift perturbations at each blocker concentration were analyzed using the CcpNmr software (CCPN Charter, Cambridge, UK) [45] and the plots of chemical shift changes (see Figure 1B) were made based on ^1^H and ^15^N shifts using the equation [(δH)^2^ + (δN/5)^2^]^1/2^ [46].

### 4.3. Cells and Viruses

The hepatoma cell line (Huh7.5.1) was maintained in complete Dulbecco’s modified Eagle medium (DMEM) supplemented with 10% fetal calf serum, 10 mM HEPES buffer, 100 U/mL penicillin, and 100 mg/mL streptomycin as described previously [35]. HCV (JFH1 strain with adaptive mutations) was amplified, and the titers were determined as described previously [47]. The virus was purified and stored in Thermo Scientific Revco ULT. All the experiments involving HCV were performed in BSL-2 facility at Institut Pasteur of Shanghai following the regulations.

### 4.4. Cell Viability and Cytotoxicity Assays

Cell viability and cytotoxicity were detected by CellTiter-Glo^®^ Luminescent Cell Viability Assay reagent (Promega GS7570, Madison, WI, USA). Briefly, Huh7.5.1 cells were seeded at a density of 80,000 cells per well in 24-well microplates and allowed to attach and grow overnight. The next day, the cell-culture media with serially diluted compounds in 0.5% DMSO or mock were added to the cells. After 12 h of incubation, cells were collected and lysed for the cell cytotoxicity assay.

### 4.5. Indirect Immunofluorescence

Intracellular immunostaining of HCV-infected cells was performed as described previously [47]. Briefly, the virus-infected cells were fixed, and stained with a mouse monoclonal anti-HCV NS5A antibody. Bound primary antibodies were detected by using Alexa Fluor 555-conjugated secondary antibodies (Molecular Probes, Eugene, OR, USA). Nuclei were stained with Hoechst dye.

### 4.6. Treatment of Antiviral Compounds

Huh7.5.1 cells were seeded in 24-well plates at a density of 80,000 cells per well overnight, and HCV infected the cells with MOI = 2 for 24 h. The supernatant with virus was removed and the cells were washed with PBS for 3 times. Then incubated with serially diluted compounds in 0.5% DMSO for 12 h. The supernatant was collected, and the virus production was determined by focus-reduction assay. Dose-response curves and the half maximal effective concentrations (IC50) of each compound were generated by non-linear fitting. IC50 was calculated as the concentration of inhibitor required for a 50% reduction in the HCV production.

### 4.7. Molecular Dynamics Simulation

The MD simulations were performed using the Desmond package [37] and the force field OPLS2005 [38] in a bilayer with appropriate number of counter ions to balance the net charge of the system solvated in 0.1 M NaCl. The membrane localization of the p7 (5a) hexameric complex was defined using the Orientations of Proteins in Membranes (OPM) database [48]. The protein and ligand molecules were inserted into POPC (1-palmitoyl-2-oleoyl-sn-glycero-3-phosphocholine) bilayer containing the explicit simple point charge (SPC) waters. Nose-Hoover temperature coupling [49] and Martina-Tobias-Klein method [50,51] with isotropic scaling were used to control the simulation temperature (300 K) and atmospheric pressure (1 atm). The particle-mesh Ewald (PME) method [52] was used to calculate long-range electrostatic interactions with grid spacing of 0.8 Å. Van der Waals (VDW) and short-range electrostatic interactions were smoothly truncated at 9.0 Å. Before MD simulations, the system was equilibrated using the default membrane relax protocol provided in Desmond, which consists of a series of restrained minimizations and heating process that are designed to slowly relax the system without deviating too much from the initial protein coordinates. The initial p7 (5a) channel coordinates for the MD simulation calculations were taken from Protein Data Bank [53] (PDB ID: 2M6X). After 2 ns system minimization and relaxation, the system was subject to 50 ns of normal pressure and temperature (NPT) production simulation which the configuration was saved every 50 ps.

### 4.8. Protein Modeling

The template structure of the hexameric HCV p7 channel (modified Gt5a sequence, solution NMR data in DPC-micelle, lowest-energy conformation) was obtained from the Protein Data Bank (PDB ID: 2M6X) [20]. The representative p7 sequences used were obtained from UniProtKB database for the HCV genotypes Gt1a (UniProt: P27958, residues 747-809, L44F), Gt2a (UniProt: P26660, residues 751-813), Gt3a (UniProt: Q81258, residues 753-815), Gt4a (UniProt: O39929, residues 747-809). The homology models of Gt1a, Gt2a, Gt3a, Gt4a were built from the template Gt5a (see Figure 3A,B) using Discovery Studio 4.0 software [39]. The natural sequence alignments were used. The Jalview 2.10 software [54] is used for the alignment visualization and analysis.

### 4.9. Molecular Docking

The library of all 3D ligand structures (see Tables S1 and S3) was prepared using the Discovery Studio 4.0 [39]. Their geometries were pre-optimized by molecular mechanics using steepest descent and conjugate gradient. The PDB files were converted to the PDBQT files using OpenBabel 2.2.3 software [55].

Molecular dockings of the compounds were grouped into 5 in terms of 5 genotypes (1a, 2a, 3a, 4a and 5a). The HCV p7 channel models through Gt1a to Gt5a were prepared for molecular docking using Discovery Studio 4.0 [39]. Molecular docking was performed using the Autodock Vina 1.1.2 software [40] with default scoring function parameters. The grid box size was approximately set to 22 × 30 × 62 Å in order to cover the entire binding pocket and the coordinate of grid center was set to be 22 × 9 × 9 Å. In addition, the default values were used for other parameters. The binding free energy of each protein-ligand was calculated from the built-in algorithm in Autodock Vina [40]. The best-fitted poses for each model were used for further analysis [56].

### 4.10. Chemistry

The newly-designed compounds were synthesized and used without further purification. The ^1^H NMR spectra (400 and 100 MHz, respectively) were recorded using the residual signal of deuterated solvent as internal standard. Chemical shifts are given in ppm; multiplicities are described as singlet (s), doublet (d), triplet (t), quartet (q), multiplet (m) and broad (br); coupling constants are given in Hz. The corresponding ^1^H NMR are listed in the Supplementary Information.

## 5. Conclusions

Above all, we used the NMR techniques assisted with molecular simulations to address the structure-activity relationship among all the compounds. ARD112 shows the lowest *K*d values from NMR, strongest binding affinity from simulations and the lowest IC50 from anti-HCV viral assay which could be a potential inhibitor for HCV therapy. The binding affinities for all the reported amantadine derivative compounds are in the same trends with the biological data which shows the obvious correlation between the functional groups and the efficacy. In a summary, in addition to the synergetic design that combines two parts to increase the inhibition effect, phenyl group, X-elements and ether bond are the top-3 functional groups for increasing binding affinity, thus these three types of groups could be the first choices in designing anti-p7 (HCV) drugs.

## Data Availability

The data presented in this study are available in this article or Supplementary Materials.

## Supplementary Materials

The following are available online, Figure S1: The potential p7 inhibitor ARD87 can inhibit HCV production, Figure S2: Simulation interaction diagrams of ARD87 and p7 (5a), Figure S3: The distribution of binding energies for all ligands, Figure S4: ^1^H NMR spectra of new compounds, Table S1: In-silico and in-vitro inhibitory effects of novel compounds, Table S2: ADME properties of ARD-series compounds, Table S3: In-silico binding effects of published compounds.

## Sample Availability

Samples of the ARD-series compounds are available from the authors.

## Abbreviations

HCV: hepatitis C virus; NMR: nuclear magnetic resonance; MD: molecular dynamics; DAA: direct-acting antiviral agents; HMA: hexamethylene amiloride; DHPC: 1,2-diheptanoyl-snglycero-3-phosphocholine; DPC: dodecylphosphocholine; PDB: Protein Data Bank; NOESY: nuclear Overhauser enhancement spectroscopy; EM: electron microscopy; BVDV: bovine viral diarrhea virus; ARD: amantadine or rimantadine derivatives; TROSY-HSQC: transverse relaxation optimized spectroscopy and heteronuclear single-quantum coherence; Rim: rimantadine; POPC: 1-palmitoyl-2-oleoyl-sn-glycero-3-phosphocholine; RMSD: Root Mean Square Fluctuation; RMSF: Root-Mean-Square Deviation; L-RMSF: Ligand Root Mean Square Fluctuation; QSAR: quantitative structure-activity relationship; Gt: genotype; CNBr: cyanogen bromide; DMSO: dimethyl sulphoxide; DMEM: Dulbecco’s modified Eagle medium; SPC: simple point charge; VDW: Van der Waals; NPT: normal pressure and temperature; ADME: absorption, distribution, metabolism and excretion.

## References

[1] Mohd Hanafiah K., Groeger J., Flaxman A.D., Wiersma S.T. (2013). Global epidemiology of hepatitis c virus infection: New estimates of age-specific antibody to hcv seroprevalence. Hepatology.

[2] Bacon B.R., Di Bisceglie A.M. (2001). Hepatitis c virus infection. N. Engl. J. Med..

[3] Shepard C.W., Finelli L., Alter M.J. (2005). Global epidemiology of hepatitis c virus infection. Lancet Infect. Dis..

[4] Choo Q.L., Kuo G., Weiner A.J., Overby L.R., Bradley D.W., Houghton M. (1989). Isolation of a cdna clone derived from a blood-borne non-a, non-b viral hepatitis genome. Science.

[5] Borgia S.M., Hedskog C., Parhy B., Hyland R.H., Stamm L.M., Brainard D.M., Subramanian M.G., McHutchison J.G., Mo H., Svarovskaia E. (2018). Identification of a novel hepatitis c virus genotype from Punjab, India: Expanding classification of hepatitis c virus into 8 genotypes. J. Infect. Dis..

[6] Hedskog C., Parhy B., Chang S., Zeuzem S., Moreno C., Shafran S.D., Borgia S.M., Asselah T., Alric L., Abergel A. (2019). Identification of 19 novel hepatitis c virus subtypes—Further expanding hcv classification. Open Forum Infect. Dis..

[7] de Vicente J., Hendricks R.T., Smith D.B., Fell J.B., Fischer J., Spencer S.R., Stengel P.J., Mohr P., Robinson J.E., Blake J.F. (2009). Non-nucleoside inhibitors of hcv polymerase ns5b. Part 2: Synthesis and structure-activity relationships of benzothiazine-substituted quinolinediones. Bioorg. Med. Chem. Lett..

[8] Gao M., Nettles R.E., Belema M., Snyder L.B., Nguyen V.N., Fridell R.A., Serrano-Wu M.H., Langley D.R., Sun J.H., O’Boyle D.R. (2010). Chemical genetics strategy identifies an hcv ns5a inhibitor with a potent clinical effect. Nature.

[9] Legrand-Abravanel F., Nicot F., Izopet J. (2010). New ns5b polymerase inhibitors for hepatitis c. Expert Opin. Investig. Drugs.

[10] Sofia M.J., Chang W., Furman P.A., Mosley R.T., Ross B.S. (2012). Nucleoside, nucleotide, and non-nucleoside inhibitors of hepatitis c virus ns5b rna-dependent rna-polymerase. J. Med. Chem..

[11] Aghemo A., De Francesco R. (2013). New horizons in hepatitis c antiviral therapy with direct-acting antivirals. Hepatology.

[12] Chemello L., Cavalletto L., Noventa F., Bonetti P., Casarin C., Bernardinello E., Pontisso P., Donada C., Casarin P., Belussi F. (1995). Predictors of sustained response, relapse and no response in patients with chronic hepatitis c treated with interferon-alpha. J. Viral Hepat..

[13] Enomoto N., Sakuma I., Asahina Y., Kurosaki M., Murakami T., Yamamoto C., Ogura Y., Izumi N., Marumo F., Sato C. (1996). Mutations in the nonstructural protein 5a gene and response to interferon in patients with chronic hepatitis c virus 1b infection. N. Engl. J. Med..

[14] Lin C., Lindenbach B.D., Pragai B.M., McCourt D.W., Rice C.M. (1994). Processing in the hepatitis c virus e2-ns2 region: Identification of p7 and two distinct e2-specific products with different c termini. J. Virol..

[15] Selby M.J., Glazer E., Masiarz F., Houghton M. (1994). Complex processing and Protein:Protein interactions in the e2:Ns2 region of hcv. Virology.

[16] Griffin S.D.C., Beales L.P., Clarke D.S., Worsfold O., Evans S.D., Jaeger J., Harris M.P.G., Rowlands D.J. (2003). The p7 protein of hepatitis c virus forms an ion channel that is blocked by the antiviral drug, amantadine. FEBS Lett..

[17] Qi H., Chu V., Wu N.C., Chen Z., Truong S., Brar G., Su S.Y., Du Y., Arumugaswami V., Olson C.A. (2017). Systematic identification of anti-interferon function on hepatitis c virus genome reveals p7 as an immune evasion protein. Proc. Natl. Acad. Sci. USA.

[18] Filskov J., Andersen P., Agger E.M., Bukh J. (2019). Hcv p7 as a novel vaccine-target inducing multifunctional cd4(+) and cd8(+) t-cells targeting liver cells expressing the viral antigen. Sci. Rep..

[19] Chen W., Dev J., Mezhyrova J., Pan L., Piai A., Chou J.J. (2018). The unusual transmembrane partition of the hexameric channel of the hepatitis c virus. Structure.

[20] OuYang B., Xie S., Berardi M.J., Zhao X., Dev J., Yu W., Sun B., Chou J.J. (2013). Unusual architecture of the p7 channel from hepatitis c virus. Nature.

[21] Clarke D., Griffin S., Beales L., Gelais C.S., Burgess S., Harris M., Rowlands D. (2006). Evidence for the formation of a heptameric ion channel complex by the hepatitis c virus p7 protein in vitro. J. Biol. Chem..

[22] Pavlović D., Neville D.C., Argaud O., Blumberg B., Dwek R.A., Fischer W.B., Zitzmann N. (2003). The hepatitis c virus p7 protein forms an ion channel that is inhibited by long-alkyl-chain iminosugar derivatives. Proc. Natl. Acad. Sci. USA.

[23] Premkumar A., Wilson L., Ewart G.D., Gage P.W. (2004). Cation-selective ion channels formed by p7 of hepatitis c virus are blocked by hexamethylene amiloride. FEBS Lett..

[24] Steinmann E., Penin F., Kallis S., Patel A.H., Bartenschlager R., Pietschmann T. (2007). Hepatitis c virus p7 protein is crucial for assembly and release of infectious virions. PLoS Pathog..

[25] Luscombe C.A., Huang Z., Murray M.G., Miller M., Wilkinson J., Ewart G.D. (2010). A novel hepatitis c virus p7 ion channel inhibitor, bit225, inhibits bovine viral diarrhea virus in vitro and shows synergism with recombinant interferon-alpha-2b and nucleoside analogues. Antivir. Res..

[26] Pavlovic D., Fischer W., Hussey M., Durantel D., Durantel S., Branza-Nichita N., Woodhouse S., Dwek R.A., Zitzmann N. (2005). Long alkylchain iminosugars block the hcv p7 ion channel. Adv. Exp. Med. Biol..

[27] StGelais C., Tuthill T.J., Clarke D.S., Rowlands D.J., Harris M., Griffin S. (2007). Inhibition of hepatitis c virus p7 membrane channels in a liposome-based assay system. Antivir. Res..

[28] Montserret R., Saint N., Vanbelle C., Salvay A.G., Simorre J.P., Ebel C., Sapay N., Renisio J.G., Bockmann A., Steinmann E. (2010). Nmr structure and ion channel activity of the p7 protein from hepatitis c virus. J. Biol. Chem..

[29] Luik P., Chew C., Aittoniemi J., Chang J., Wentworth P., Dwek R.A., Biggin P.C., Venien-Bryan C., Zitzmann N. (2009). The 3-dimensional structure of a hepatitis c virus p7 ion channel by electron microscopy. Proc. Natl. Acad. Sci. USA.

[30] Zhao L., Wang S., Du L., Dev J., Zhou L., Liu Z., Chou J.J., OuYang B. (2016). Structural basis of interaction between the hepatitis c virus p7 channel and its blocker hexamethylene amiloride. Protein Cell.

[31] Dev J., Bruschweiler S., Ouyang B., Chou J.J. (2015). Transverse relaxation dispersion of the p7 membrane channel from hepatitis c virus reveals conformational breathing. J. Biomol. NMR.

[32] Shiryaev V.A., Radchenko E.V., Palyulin V.A., Zefirov N.S., Bormotov N.I., Serova O.A., Shishkina L.N., Baimuratov M.R., Bormasheva K.M., Gruzd Y.A. (2018). Molecular design, synthesis and biological evaluation of cage compound-based inhibitors of hepatitis c virus p7 ion channels. Eur. J. Med. Chem..

[33] Mandour Y.M., Breitinger U., Ma C., Wang J., Boeckler F.M., Breitinger H.G., Zlotos D.P. (2018). Symmetric dimeric adamantanes for exploring the structure of two viroporins: Influenza virus m2 and hepatitis c virus p7. Drug Des. Dev. Ther..

[34] Kalita M.M., Griffin S., Chou J.J., Fischer W.B. (2015). Genotype-specific differences in structural features of hepatitis c virus (hcv) p7 membrane protein. Biochim. Biophys. Acta.

[35] Griffin S., Stgelais C., Owsianka A.M., Patel A.H., Rowlands D., Harris M. (2008). Genotype-dependent sensitivity of hepatitis c virus to inhibitors of the p7 ion channel. Hepatology.

[36] Shaw J., Gosain R., Kalita M.M., Foster T.L., Kankanala J., Mahato D.R., Abas S., King B.J., Scott C., Brown E. (2020). Rationally derived inhibitors of hepatitis c virus (hcv) p7 channel activity reveal prospect for bimodal antiviral therapy. eLife.

[37] Bowers K., Chow E., Xu H., Dror R.O., Eastwood M.P., Gregersen B.A., Klepeis J.L., Kolossvary I.N., Moraes M.A., Sacerdoti F.D. Scalable algorithms for molecular dynamics simulations on commodity clusters. Proceedings of the 2006 ACM/IEEE Conference on Supercomputing (SC’06).

[38] Jorgensen W.L., Tirado-Rives J. (1988). The opls (optimized potentials for liquid simulations) potential functions for proteins, energy minimizations for crystals of cyclic peptides and crambin. J. Am. Chem. Soc..

[39] Jason B. Dassault Systèmes Biovia, Discovery Studio Modeling Environment, Release 4.0, San Diego: Dassault Systèmes. https://www.3dsbiovia.com/.

[40] Trott O., Olson A.J. (2010). Autodock vina: Improving the speed and accuracy of docking with a new scoring function, efficient optimization, and multithreading. J. Comput. Chem..

[41] Zanaga L.P., Miotto N., Mendes L.C., Stucchi R.S.B., Vigani A.G. (2016). Treatment of hepatitis c virus genotype 3 infection with direct-acting antiviral agents. Braz. J. Med. Biol. Res..

[42] Foster T.L., Verow M., Wozniak A.L., Bentham M.J., Thompson J., Atkins E., Weinman S.A., Fishwick C., Foster R., Harris M. (2011). Resistance mutations define specific antiviral effects for inhibitors of the hepatitis c virus p7 ion channel. Hepatology.

[43] Mihm U., Grigorian N., Welsch C., Herrmann E., Kronenberger B., Teuber G., von Wagner M., Hofmann W.P., Albrecht M., Lengauer T. (2006). Amino acid variations in hepatitis c virus p7 and sensitivity to antiviral combination therapy with amantadine in chronic hepatitis c. Antivir. Ther..

[44] Delaglio F., Grzesiek S., Vuister G.W., Zhu G., Pfeifer J., Bax A. (1995). Nmrpipe: A multidimensional spectral processing system based on unix pipes. J. Biomol. NMR.

[45] Vranken W.F., Boucher W., Stevens T.J., Fogh R.H., Pajon A., Llinas M., Ulrich E.L., Markley J.L., Ionides J., Laue E.D. (2005). The ccpn data model for nmr spectroscopy: Development of a software pipeline. Proteins.

[46] Schumann F.H., Riepl H., Maurer T., Gronwald W., Neidig K.P., Kalbitzer H.R. (2007). Combined chemical shift changes and amino acid specific chemical shift mapping of protein-protein interactions. J. Biomol. NMR.

[47] Zhong J., Gastaminza P., Cheng G., Kapadia S., Kato T., Burton D.R., Wieland S.F., Uprichard S.L., Wakita T., Chisari F.V. (2005). Robust hepatitis c virus infection in vitro. Proc. Natl. Acad. Sci. USA.

[48] Lomize M.A., Lomize A.L., Pogozheva I.D., Mosberg H.I. (2006). Opm: Orientations of proteins in membranes database. Bioinformatics.

[49] Hoover W.G. (1985). Canonical dynamics: Equilibrium phase-space distributions. Phys. Rev. A Gen. Phys..

[50] Martyna G.J., Tobias D.J., Klein M.L. (1994). Constant Pressure Molecular Dynamics Algorithms. J. Chem. Phys..

[51] Deng Z., Martyna G.J., Klein M.L. (1992). Structure and dynamics of bipolarons in liquid ammonia. Phys. Rev. Lett..

[52] Petersen H.G. (1995). Accuracy and Efficiency of the Particle Mesh Ewald Method. J. Chem. Phys..

[53] Berman H.M., Westbrook J.D., Feng Z., Gilliland G.L., Bhat T.N., Weissig H., Shindyalov I.N., Bourne P.E. (2000). The Protein Data Bank. Nucleic Acids Res..

[54] Waterhouse A.M., Procter J.B., Martin D.M., Clamp M., Barton G.J. (2009). Jalview version 2—A multiple sequence alignment editor and analysis workbench. Bioinformatics.

[55] O’Boyle N.M., Banck M., James C.A., Morley C., Vandermeersch T., Hutchison G.R. (2011). Open babel: An open chemical toolbox. J. Cheminform..

[56] PyMOL0.99rc6. Delano Scientific llc. http://www.pymol.org/.

