# Supplementary material for: Inhibitor Development against p7 Channel in Hepatitis C Virus
Source: Molecules. 2021 Mar 3;26(5):1350. doi: 10.3390/molecules26051350 (PMC7961618; doi:10.3390/molecules26051350)
Supplement: Supplementary file 1 [file molecules-26-01350-s001.pdf]

Supporting Information

Keywords: rational design, inhibitors, docking, MD, p7, HCV production

Authors: Shukun Wei[1,2#], Xiaoyou Hu[2,3#], Lingyu Du[1], Linlin Zhao[1,4], Hongjuan Xue[5], Chaolun Liu[6], James J. Chou[4], Jin Zhong[2,3,6], Yimin Tong[3*], Shuqing Wang[7,*], Bo OuYang[1,2*]

Author Affiliations:

1. State Key Laboratory of Molecular Biology, Center for Excellence in Molecular Cell Science, Shanghai Institute of Biochemistry and Cell Biology, Chinese Academy of Sciences; University of Chinese Academy of Sciences, 333 Haike Road, Shanghai 201203, China; ouyang@sibcb.ac.cn (B.O.); weishukun2017@sibcb.ac.cn (S. W.); dulingyu@sibcb.ac.cn (L.D.)

2. University of Chinese Academy of Sciences, Beijing, 100049, China; xyhu@ips.ac.cn (X. H.)

3. CAS Key Laboratory of Molecular Virology and Immunology, Institute Pasteur of Shanghai, Chinese Academy of Sciences, Shanghai, 200031, China; jzhong@ips.ac.cn (J. Z.)

4. Department of Biological Chemistry and Molecular Pharmacology, Harvard Medical School, Boston, MA 02115, USA; lzhao@crystal.harvard.edu (L.Z.); james_chou@hms.harvard.edu (J.J.C.)

5. National Facility for Protein Science in Shanghai, ZhangJiang lab, Shanghai Advanced Research Institute, Chinese Academy of Sciences, Shanghai 201210, China; xuehongjuan@sari.ac.cn (H. X.)

6. ShanghaiTech University, Shanghai 201210, China; clliu@ips.ac.cn (C. L.)

7. School of Pharmacy, Tianjin Medical University, Tianjin 300070, China; wangshuqing@tmu.edu.cn (S.W)

* Correspondence: ymtong@sibs.ac.cn (Y.T.); wangshuqing@tmu.edu.cn (S.W); ouyang@sibcb.ac.cn (B.O.)

1. Supplementary tables

Table S1. *In-silico* and *in-vitro* inhibitory effects of novel compounds.

Ligand Name	Ligand[a]	*Kd* (μM) GT5a[b]	Binding Affinity (kcal/mol)[c]				
			GT1a	GT2a	GT3a	GT4a	GT5a
Rimantadine	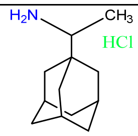	98	−6.1	−5.6	−5.9	−5.7	−6.2
ARD1	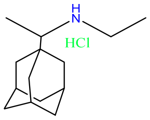	487	−5.5	−5.6	−5.5	−6	−5.1
ARD2	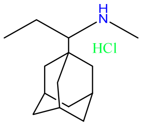	334	−5.6	−5.7	−5.2	−5.2	−5.3
ARD6	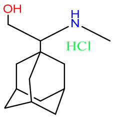	344	−5.4	−5.8	−5.4	−5.7	−5.2
ARD8	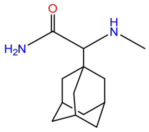	707	−5.4	−5.8	−5.4	−5.6	−5.3
ARD9	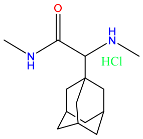	418	−6	−6.1	−5.7	−6	−5.7

ARD10	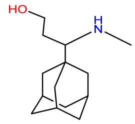	932	−5.2	−5.8	−5.3	−5.2	−5.1
ARD13	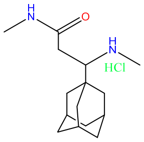	414	−6.2	−6.6	−5.7	−5.9	−5.7
ARD14	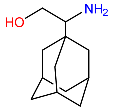	269	−6.1	−5.9	−5.6	−6.6	−5.4
ARD15	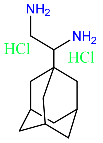	195	−5.7	−6	−5.5	−5.4	−5.4
ARD16	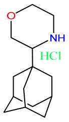	150	−6.7	−6.9	−5.9	−6.1	−6.4
ARD18	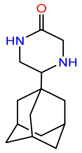	588	−6.1	−6.6	−6.3	−6.3	−6.1
ARD19	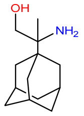	333	−5.5	−5.9	−5.3	−5.6	−5.3
ARD20	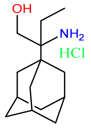	436	−5.6	−6.7	−5.9	−5.9	−5.7
ARD22	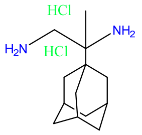	285	−5.6	−5.8	−5.7	−5.8	−5.4
ARD23	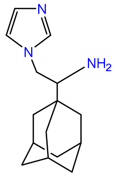	2453	−6.1	−6.3	−5.9	−6.1	−5.8
ARD24	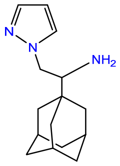	866	−6	−6.7	−5.8	−6.2	−5.8
ARD25	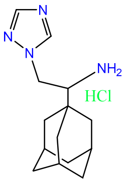	7530	−6.2	−6.4	−5.7	−6.1	−6

ARD33	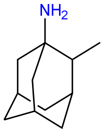	3209	−5.1	−5.3	−5.1	−5.2	−4.9
ARD37	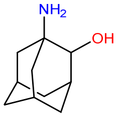	332	−5.2	−5.9	−5.3	−5.3	−5.3
ARD39	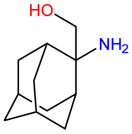	327	−5.5	−5.9	−5.8	−5.6	−5.3
ARD40	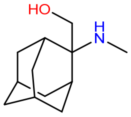	677	−5	−5.6	−5.1	−5.6	−5
ARD41	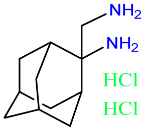	3298	−4.8	−5.4	−5	−5.4	−4.9
ARD42	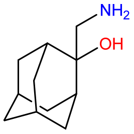	3077	−4.9	−5.6	−5.3	−5.4	−5
ARD49	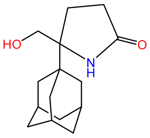	276	−6.3	−6.6	−6.1	−6.4	−5.9
ARD50	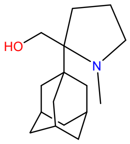	821	−6.1	−6.1	−5.6	−5.8	−6.1
ARD51	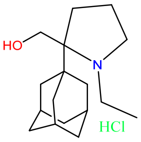	477	−6	−6.1	−5.5	−6	−5.8
ARD58	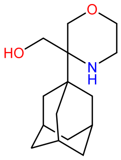	173	−6.5	−6.8	−5.8	−6.3	−6.5
ARD66	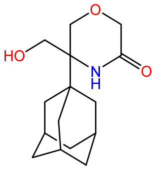	128	−6.5	−6.6	−6.4	−6.7	−6.6
ARD68	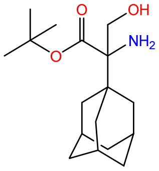	202	−6.4	−6.6	−6	−6.3	−6.1

ARD71	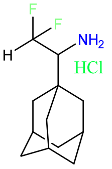	2458	−5.6	−6	−5.6	−5.9	−5.4
ARD80	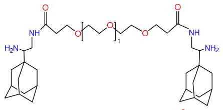	102	−7.2	−5.7	−6.6	−6.3	−6.6
ARD81	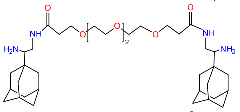	81	−7.2	−7.2	−7.2	−6.9	−6.7
ARD83	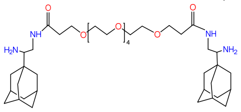	70	−7.3	−7.5	−7.2	−6.9	−6.7
ARD84	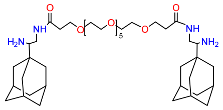	58	−6.6	−7.7	−7.2	−7	−7
ARD87	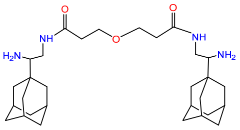	43	−9.1	−7.9	−7.3	−7	−7.5
ARD88	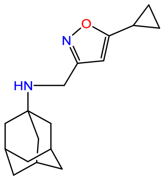	397	−6.7	−7	−6.5	−6.5	−6.8
ARD89	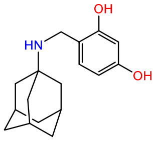	112	−7.2	−7.1	−7.2	−7	−6.9
ARD98	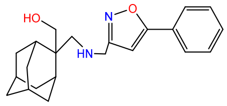	102	−7.9	−7.6	−7.4	−7.1	−7.6
ARD99	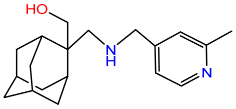	852	−6.4	−6.8	−6.2	−5.9	−6.6
ARD100	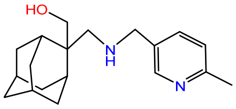	107	−7	−8.1	−7.4	−6.3	−6.9
ARD101	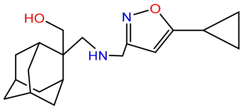	105	−7.4	−8.2	−7.6	−6.8	−7.2
ARD112	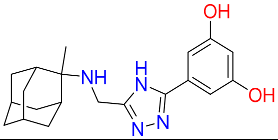	5	−8.5	−9.2	−8.5	−8.4	−9.4

[a] The ligands used above are newly designed and synthesized except rimantadine.

[b] The *Kd* values were calculated from the NMR titrations of p7 (5a).

[c] The binding affinities were newly calculated using ARD-series compounds.

Table S2. ADME properties of ARD-series compounds

Ligand Name	Ligand	MW[a]	log*P*[b]	log*S*[c]	PSA[d]	H-don[e]	H-acc[f]
ARD1	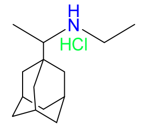	243.8	2.17	−3.71	16.61	1	0
ARD2	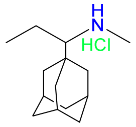	243.8	2.17	−3.58	16.61	1	0
ARD6	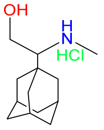	245.78	0.76	−2.85	36.84	2	1
ARD8	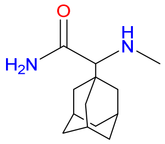	222.33	0.25	−3.33	59.7	2	1
ARD9	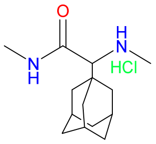	272.8	0.51	−3.22	45.71	2	1
ARD10	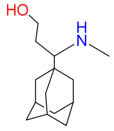	223.35	1.15	−3.05	36.84	2	1
ARD13	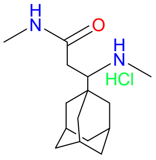	286.83	0.9	−3.11	45.71	2	1
ARD14	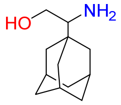	195.3	0.81	−2.77	47.87	2	1
ARD15	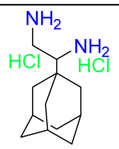	267.22	0.06	−2.64	55.28	2	0

ARD16	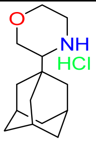	257.79	1.16	−3.34	25.84	1	1
ARD18	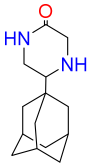	234.34	0.26	−3.37	45.71	2	1
ARD19	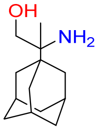	209.33	1.2	−3.1	47.87	2	1
ARD20	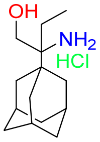	259.8	1.59	−3.3	47.87	2	1
ARD22	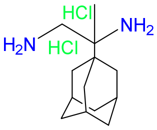	281.2	0.45	−2.97	55.28	2	0
ARD23	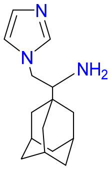	245.36	1.98	−3.27	45.46	1	1
ARD24	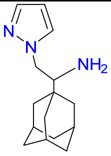	245.36	1.98	−3.1	45.46	1	1
ARD25	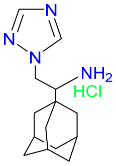	282.8	1.37	−3.26	58.35	1	2
ARD33	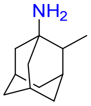	165.28	1.44	−2.29	27.64	1	0
ARD37	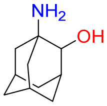	167.25	0.17	−1.05	47.87	2	1
ARD39	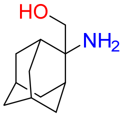	181.27	0.42	−1.76	47.87	2	1

ARD40	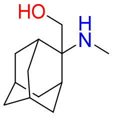	195.3	0.37	−1.83	36.84	2	1
ARD41	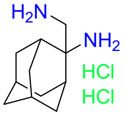	253.2	−0.33	−1.63	55.28	2	0
ARD42	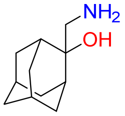	181.3	0.42	−1.76	47.87	2	1
ARD49	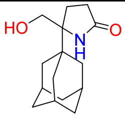	249.4	1.84	−3.51	49.33	2	2
ARD50	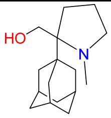	249.4	1.24	−3.53	24.67	2	1
ARD51	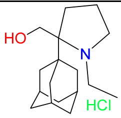	299.7	1.63	−3.86	24.67	2	1
ARD58	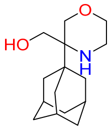	251.4	0.53	−3.14	46.07	2	2
ARD66	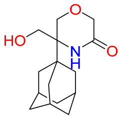	265.4	1.08	−3.57	58.56	2	3
ARD68	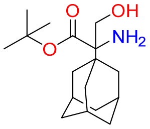	295.4	1.52	−4.16	74.17	2	2
ARD71	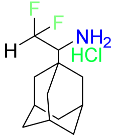	251.74	2.08	−3.5	27.64	1	0
ARD80	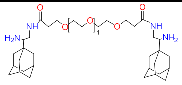	602.85	1.7	−6.9	141.17	4	5
ARD81	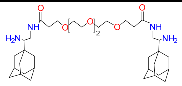	646.9	1.72	−7.04	150.4	4	6
ARD83	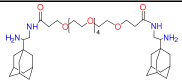	735.01	1.75	−7.32	168.86	4	8

ARD84	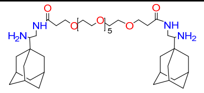	779.06	0.29	−7.1	153.43	4	9
ARD87	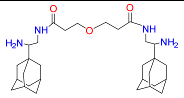	514.74	1.67	−6.61	122.71	4	3
ARD88	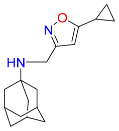	272.39	2.85	−3.34	42.64	1	1
ARD89	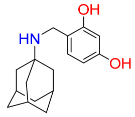	273.37	2.4	−3.2	57.07	3	2
ARD98	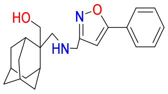	352.47	3.11	−6	62.87	2	2
ARD99	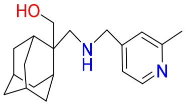	300.44	2.15	−3.86	49.73	2	2
ARD100	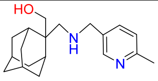	300.44	2.15	−3.86	49.73	2	2
ARD101	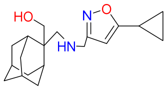	316.44	2.32	−4.22	62.87	2	2
ARD112	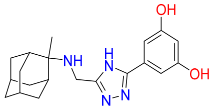	354.46	2.43	−4.52	98.64	4	4

[a]The molecular mass (MW) should be less than 500 Da

[b]The predicted octanol/water partition coefficient (− 0.4 to 5.6)

[c]The predicted aqueous solubility S (mol dm^{-3}) is the concentration of the solute in a saturated solution that is in equilibrium with the crystalline solid (− 6.5 to 0.5)

[d]The van der Waals surface area (Å^2) of polar nitrogen and oxygen atoms (7.0–200)

[e]The hydrogen-bond donors (H donor) should be no more than five

[f]The hydrogen-bond acceptors (H acceptor) should be no more than ten

Table S3. *In-silico* binding effects of published compounds

Ligand Name	Ligand [a]	Binding Affinity (kcal/mol) [b]				
		Gt1a	Gt2a	Gt3a	Gt4a	Gt5a
Amantadine	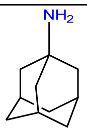	−5.5	−5.4	−5	−5.1	−4.9
Rimantadine	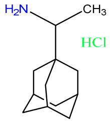	−6.1	−5.6	−5.9	−5.7	−6.2
A1	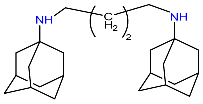	−9.1	−7.3	−7.2	−7.1	−7.7
A2	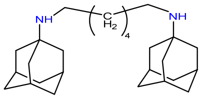	−8.7	−8	−7.3	−7.3	−7.5
A3	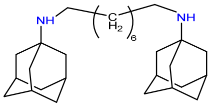	−7.9	−7.7	−6.8	−7.3	−7.5
A4	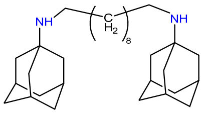	−8.1	−7.8	−6.7	−7.3	−5.9
A5	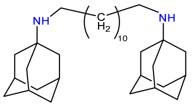	−8.6	−8.1	−6.7	−7.4	−7.6
B1	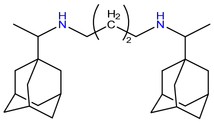	−7.6	−6.9	−7.6	−7.9	−8.2
B2	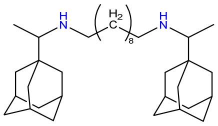	−7.5	−7.2	−7.2	−6.6	−6.8
B3	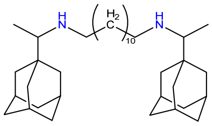	−7.8	−7.6	−7.6	−7.6	−6.7
B4	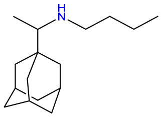	−6.5	−5.5	−5.2	−7.3	−5.7
3	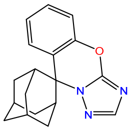	−8	−9.2	−7.7	−8.2	−8.3

8	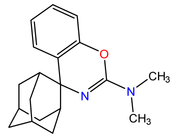	−7.8	−7.5	−6.9	−7.9	−7.3
9	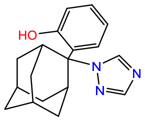	−7.5	−7.7	−6.8	−6.9	−7.4
10	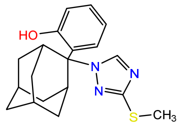	−6.9	−7.1	−6.5	−6.6	−7.2
12	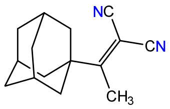	−7.1	−7.3	−6.6	−7	−6.9
13	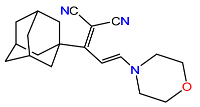	−7.7	−7.1	−7.2	−6.8	−6.5
15	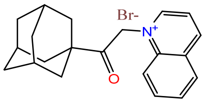	−8.1	−8.3	−7.5	−8.1	−8.2
22	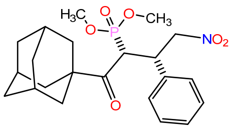	−7.7	−7	−6.6	−7.3	−6.9
24	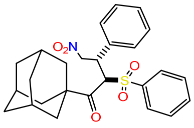	−8.8	−7.3	−7.8	−7.8	−8.1
26	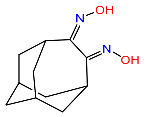	−7	−7.4	−6.6	−6.8	−6.3
32	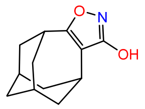	−7.2	−7.5	−6.4	−7	−6.4
33	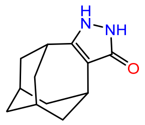	−6.9	−6.5	−6.1	−6	−6.2
36	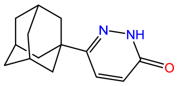	−7.4	−7.4	−7.3	−7.3	−6.7
37	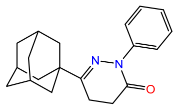	−8.6	−8.7	−7.9	−8.1	−7.9
40	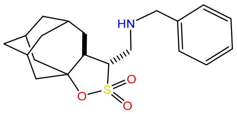	−8.2	−7.7	−8	−7.6	−8.2
48	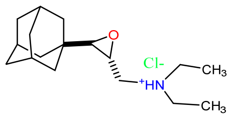	−6.6	−6.7	−5.9	−6.4	−6.1

51	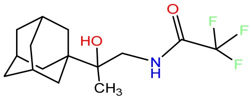	−7.9	−7.3	−7.9	−6.9	−6.8
54	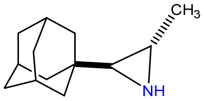	−6.2	−6.3	−5.8	−6.2	−5.8
55	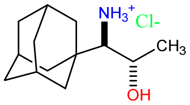	−5.9	−6.3	−5.6	−5.8	−5.8
56	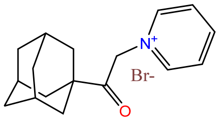	−6.6	−6.6	−6.2	−7.1	−7.1
57	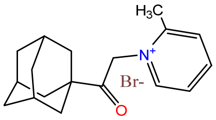	−6.5	−6.6	−6.1	−6.5	−6.8
58	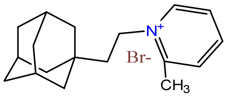	−6.6	−7.6	−6.5	−7.4	−7.4
60	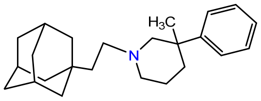	−8.5	−8.9	−8.2	−8.7	−8.7
61	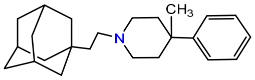	−9.4	−9.1	−8.5	−9.1	−9
62	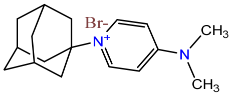	−7.8	−6.9	−6.2	−6.8	−6.8
63	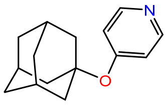	−7.2	−6.8	−5.9	−6.4	−6.3
64	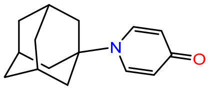	−7.4	−7	−6	−6.8	−6.3
65	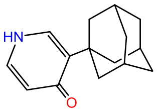	−7.8	−7.3	−6.3	−7.3	−6.6
66	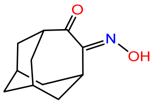	−6.6	−6	−6	−6	−5.5
68	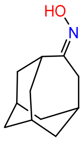	−6.3	−6.2	−5.9	−6.7	−5.8
69	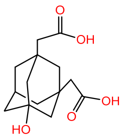	−7.3	−6.7	−6.9	−6.6	−6.2

71	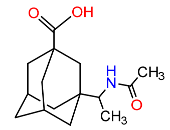	−6.7	−7.2	−6.4	−6.5	−6.7
72	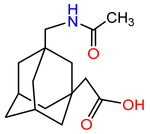	−7.2	−6.5	−6.5	−6.3	−6.1
73	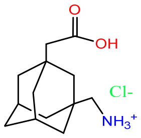	−6.8	−6.2	−5.5	−5.7	−5.9
74	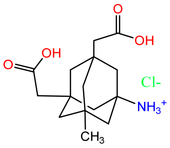	−7.3	−6.7	−6.7	−6.4	−6.2
75	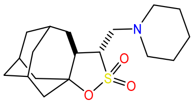	−7.6	−7.2	−6.5	−7.1	−7.6
76	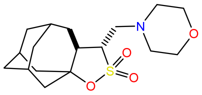	−7.7	−7.6	−7.1	−7.3	−7.7
77	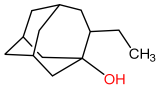	−6.5	−6.3	−6	−6.8	−6.1
78	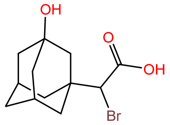	−5.8	−6.2	−5.5	−5.7	−5.4
79	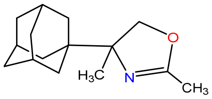	−6.6	−7.1	−6.2	−6.4	−6.5

[a] The ligands used above are from the published papers [1,2]

[b] The binding affinities were newly calculated using previously reported compounds [1,2]

2. Supplementary figures

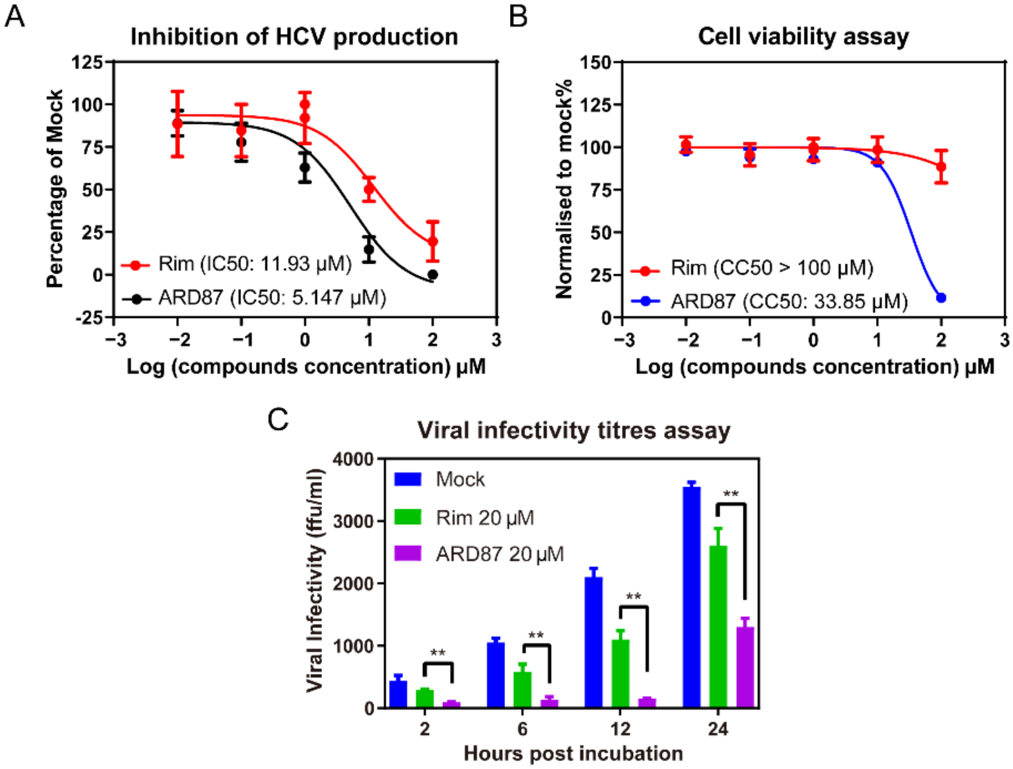

Figure S1. The potential p7 inhibitor ARD87 can inhibit HCV production. (A)The inhibition of HCV production was determined. Huh7.5.1 cells infected by JFH1 at an MOI of 2 for 1 day and were treated with a serial dilution of Rim or ARD87. 12 hours later, the viral production was detected by titration assay. (B) After incubation with each compound, the cell viability and cytotoxicity were detected by CellTiter-Glo® Luminescent Cell Viability Assay reagent (Promega GS7570, Madison, WI, USA). (C) Time course of HCV production with the treatment of ARD87 and Rim at concentration of 20 μM. Data were expressed as the percentage of mock treatment control. The error bars were calculated from three individual measurements. (** refers to $p<0.05$, ns: no significance, ARD87 compared to Rim). Two independent experiments were repeated showing similar results.

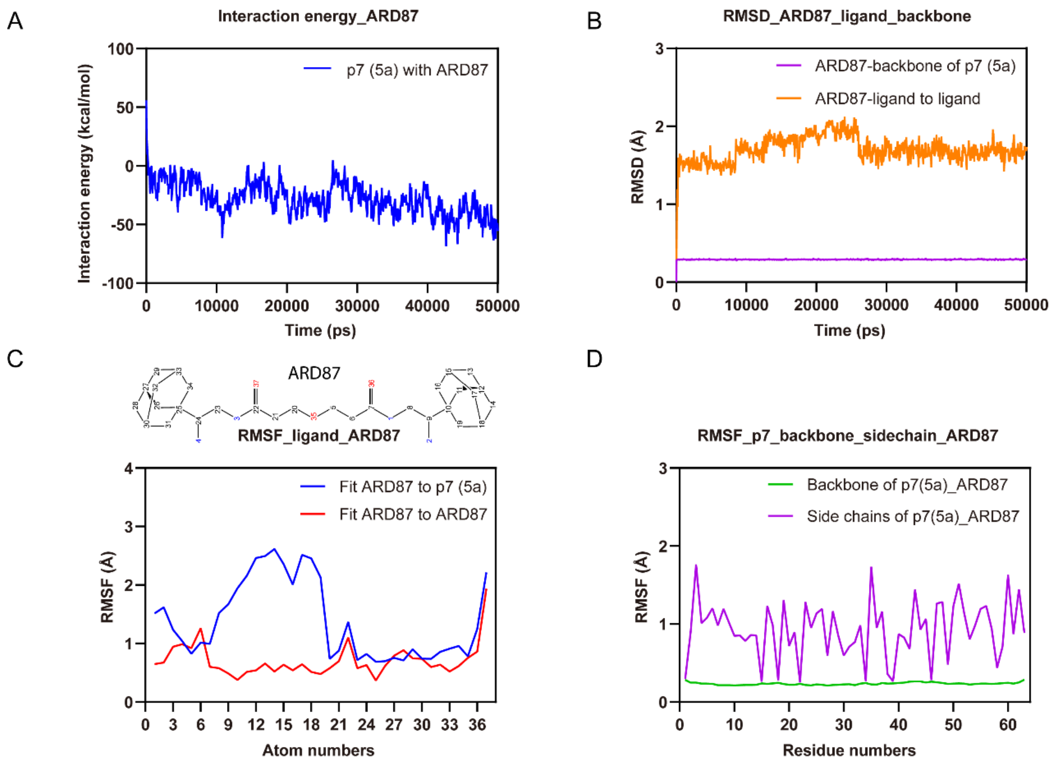

Figure S2. Simulation interaction diagrams of ARD87 and p7 (5a). (A) Interaction energies of complexes ARD87 and p7 (5a). Energies are mostly negative through the whole process for their stableness. (B) The Root Mean Square Deviation (RMSD) shows the evolution of p7 (5a) and ARD87 with the changes of the order under 1 Å. (C) The Ligand Root Mean Square Fluctuation (L-RMSF) of ARD87 shows the internal atom fluctuations of the ligand with respect to the protein p7 (5a). (D) The Root Mean Square Fluctuation (RMSF) of residues characterizes local changes along the protein chains (backbone and sidechains) for complexes of ARD87 and p7 (5a).

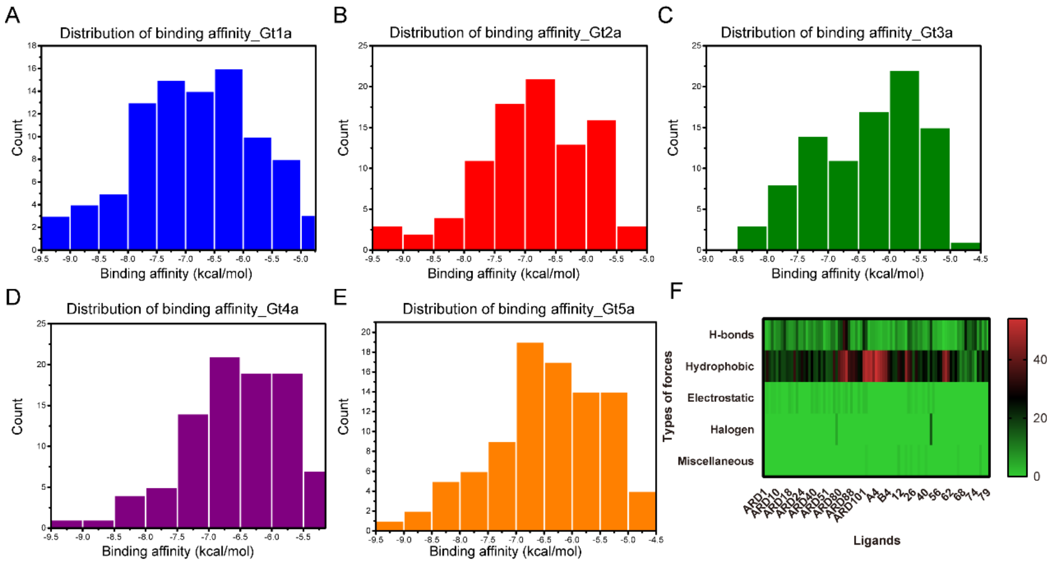

Figure S3. The distribution of binding energies for all the ligands. The binding energies to the p7 channel variants, including (A) Gt 1a, (B) Gt 2a, (C) Gt 3a, (D) Gt 4a and (E) Gt 5a. (F) Total frequence of interaction forces between all the compounds and the p7 channels of 5 genotypes, the more red, the more frequent on interactions.

Figure S4. ^{1}H NMR spectra of new compounds.

1-((3r,5r,7r)-adamantan-1-yl)-N-ethylethan-1-amine (**ARD1**)

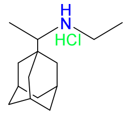

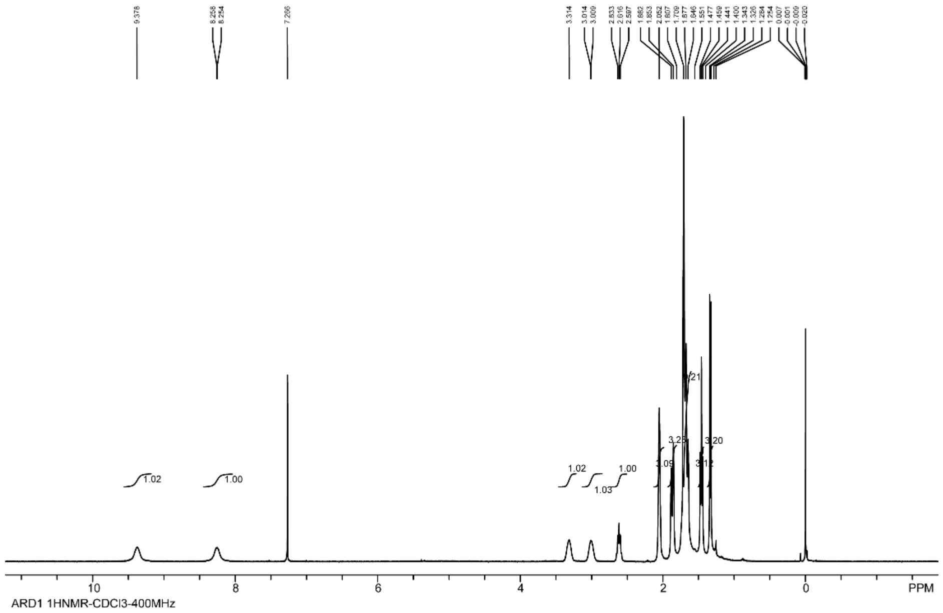

1-((3r,5r,7r)-adamantan-1-yl)-N-methylpropan-1-amine (**ARD2**)

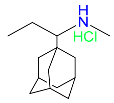

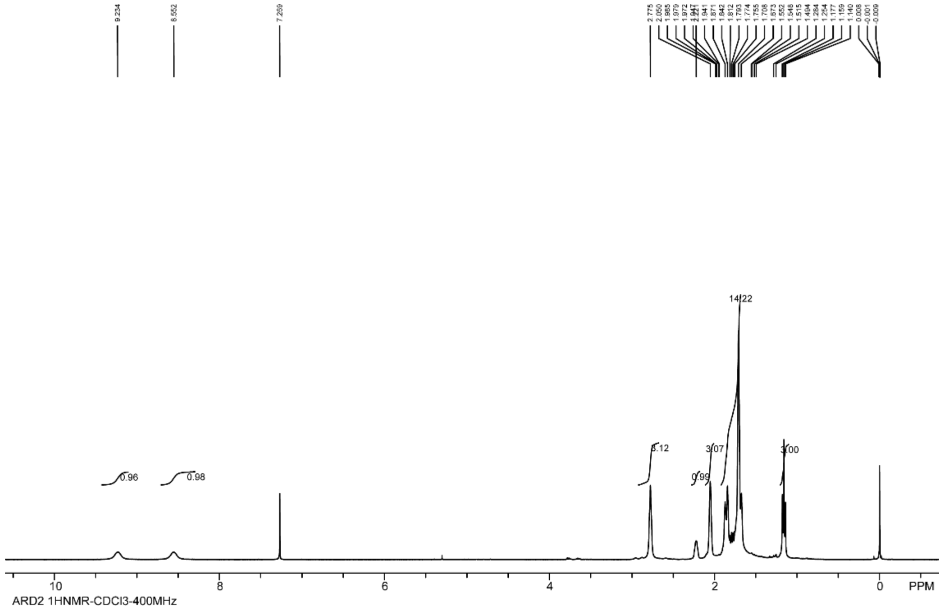

2-((3r,5r,7r)-adamantan-1-yl)-2-(methylamino)ethan-1-ol hydrochloride (**ARD6**)

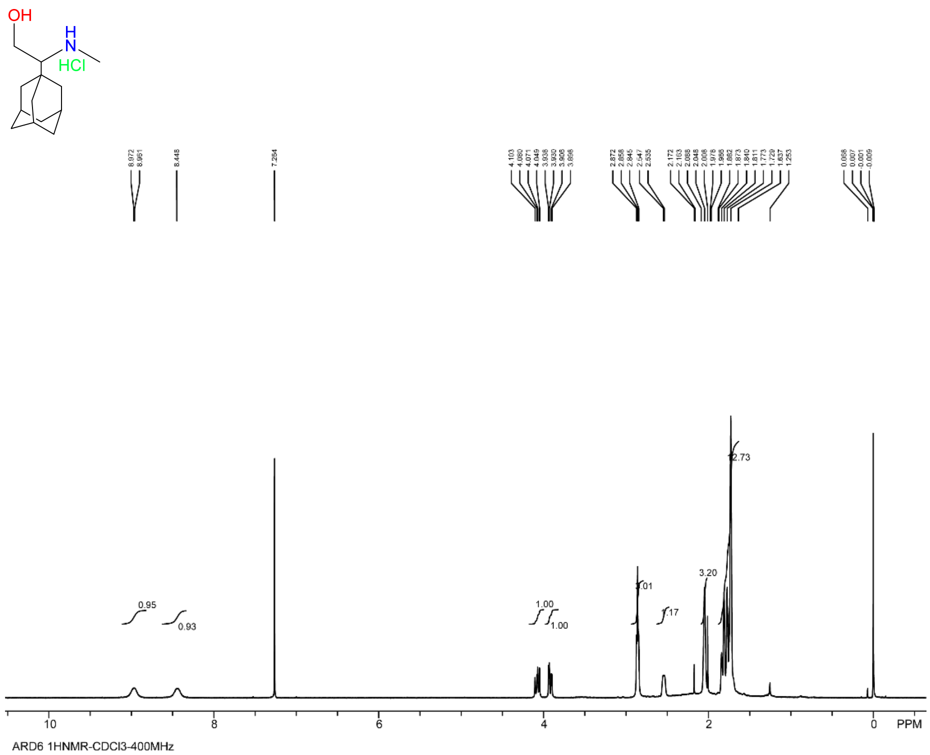

2-((3r,5r,7r)-adamantan-1-yl)-2-(methylamino)acetamide (**ARD8**)

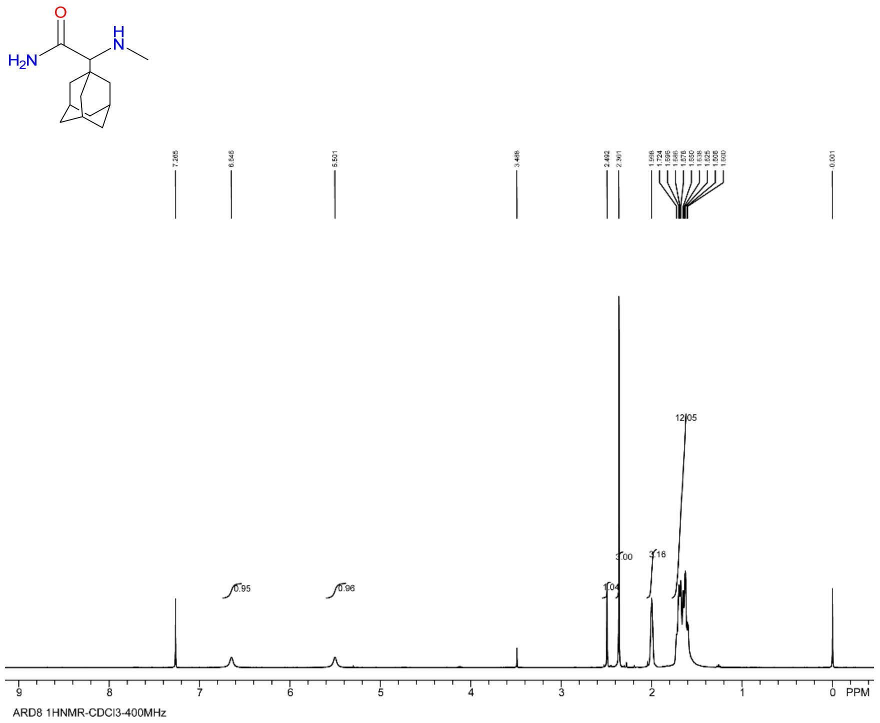

2-((3r,5r,7r)-adamantan-1-yl)-N-methyl-2-(methylamino)acetamide (**ARD9**)

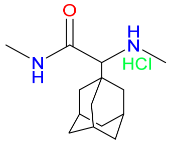

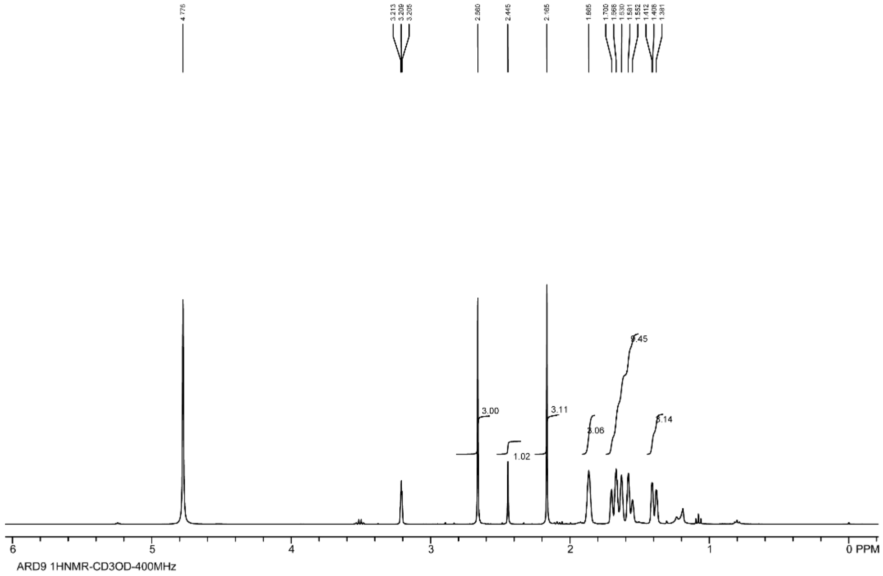

3-((3r,5r,7r)-adamantan-1-yl)-3-(methylamino)propan-1-ol (**ARD10**)

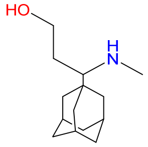

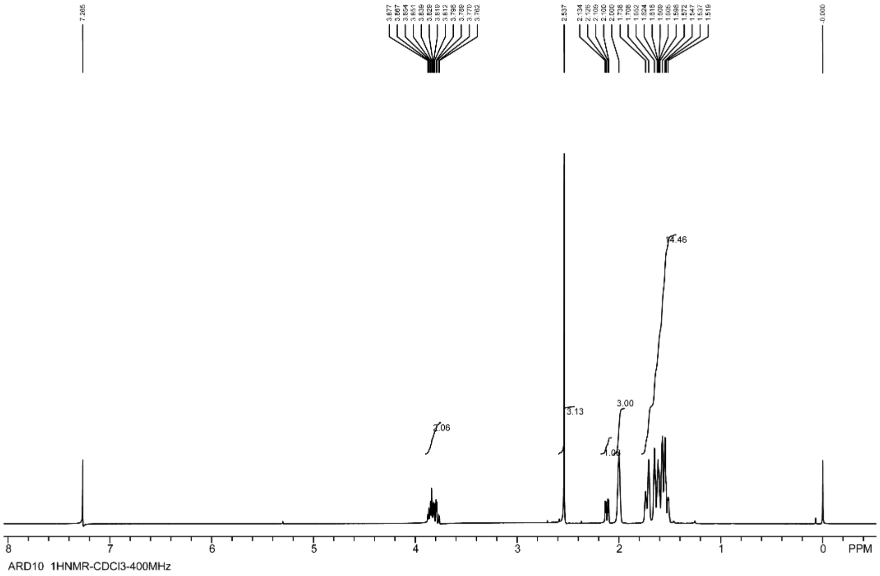

3-((3r,5r,7r)-adamantan-1-yl)-N-methyl-3-(methylamino)propanamide hydrochloride (**ARD13**)

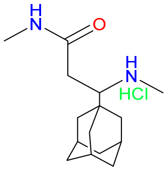

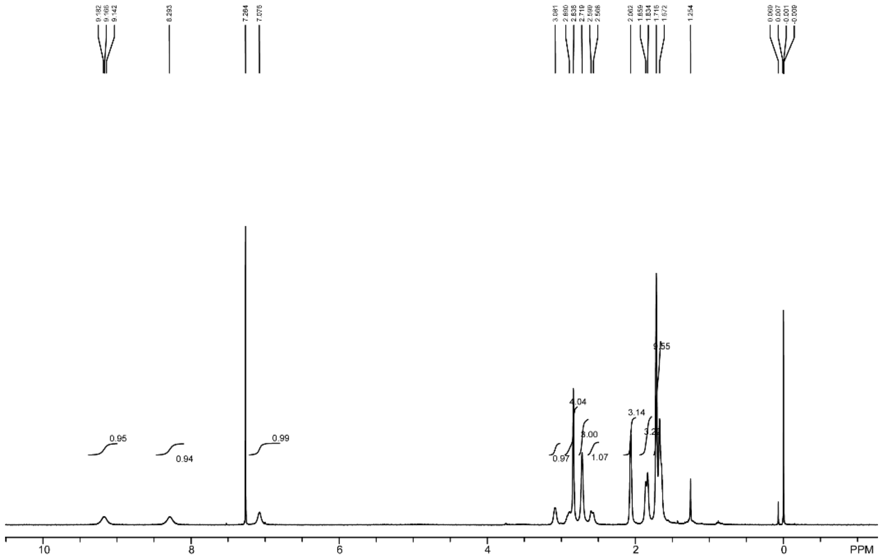

ARD13 1HNMR-CDCl3-400MHz

2-((3r,5r,7r)-adamantan-1-yl)-2-aminoethan-1-ol (**ARD14**)

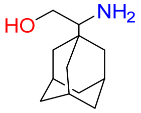

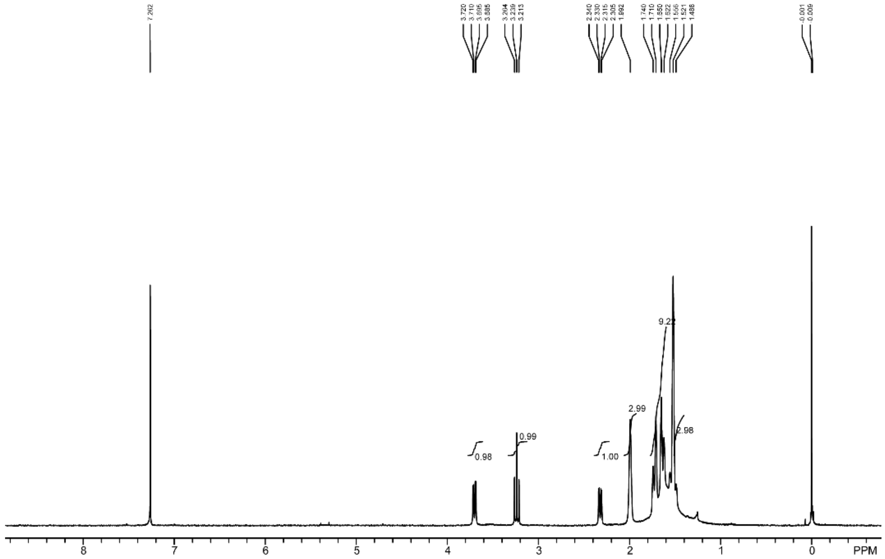

ARD14 1HNMR-CDCl3-400MHz

1-((3r,5r,7r)-adamantan-1-yl)ethane-1,2-diamine (**ARD15**)

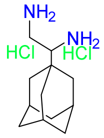

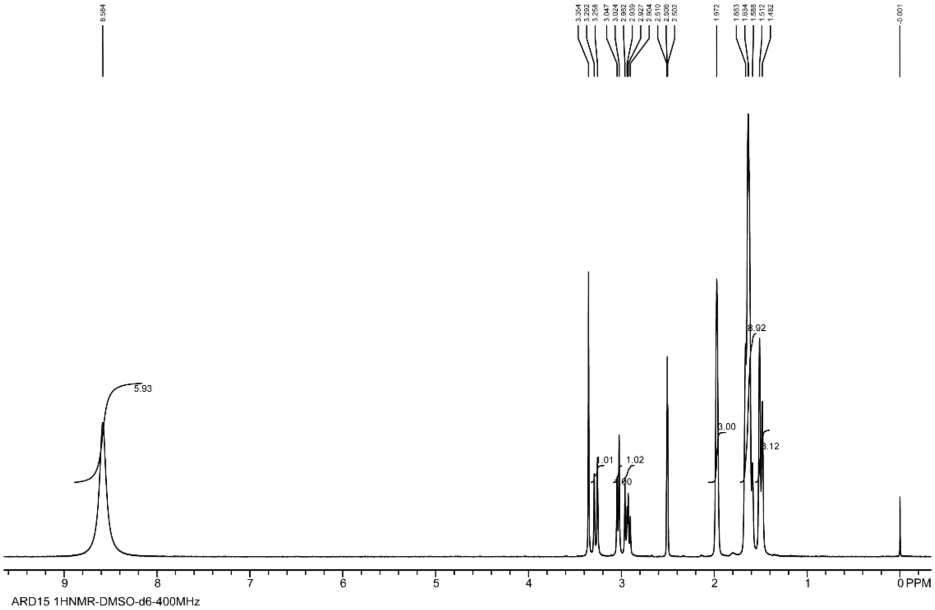

3-((3r,5r,7r)-adamantan-1-yl)morpholine (**ARD16**)

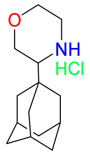

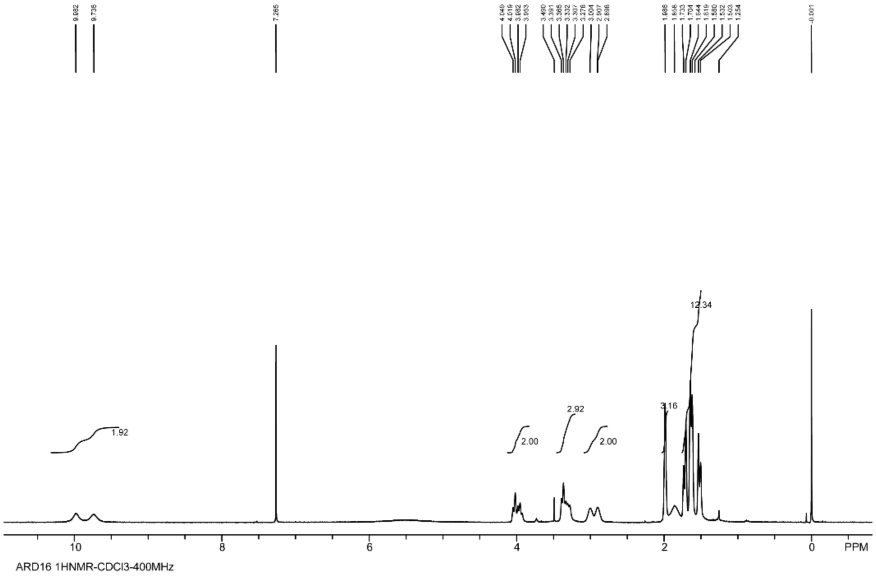

5-((3r,5r,7r)-adamantan-1-yl)piperazin-2-one (**ARD18**)

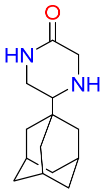

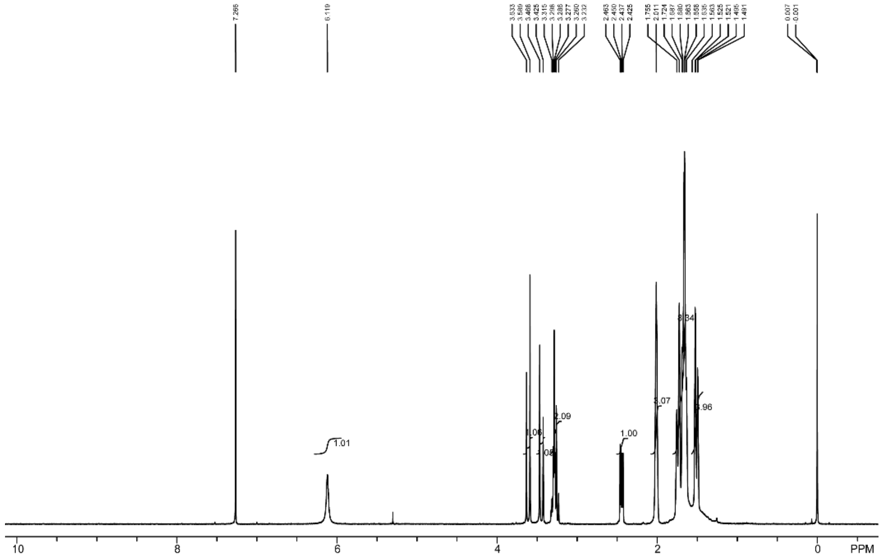

ARD18 1HNMR-CDCl3-400MHz

2-((3r,5r,7r)-adamantan-1-yl)-2-aminopropan-1-ol (**ARD19**)

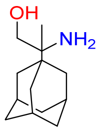

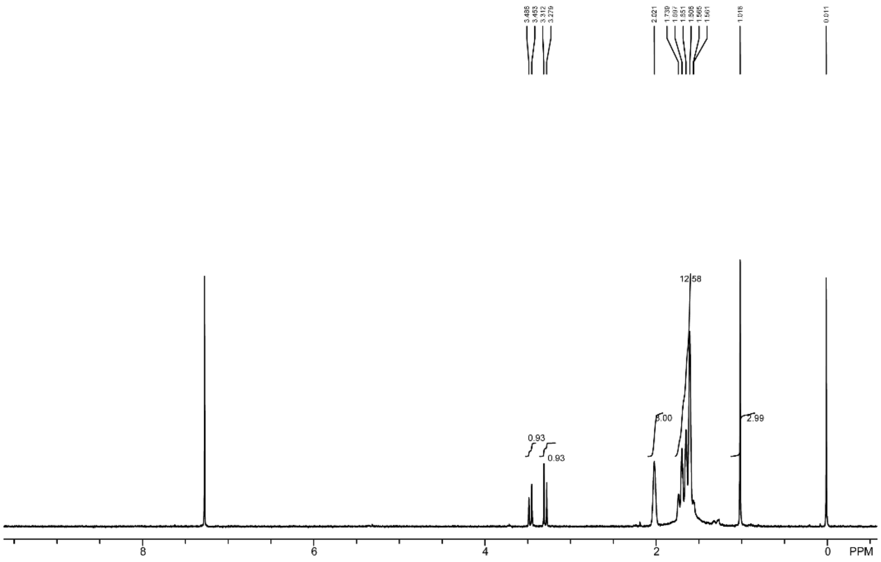

ARD19 1HNMR-CDCl3-300MHz

2-((3r,5r,7r)-adamantan-1-yl)-2-aminobutan-1-ol (**ARD20**)

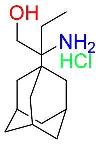

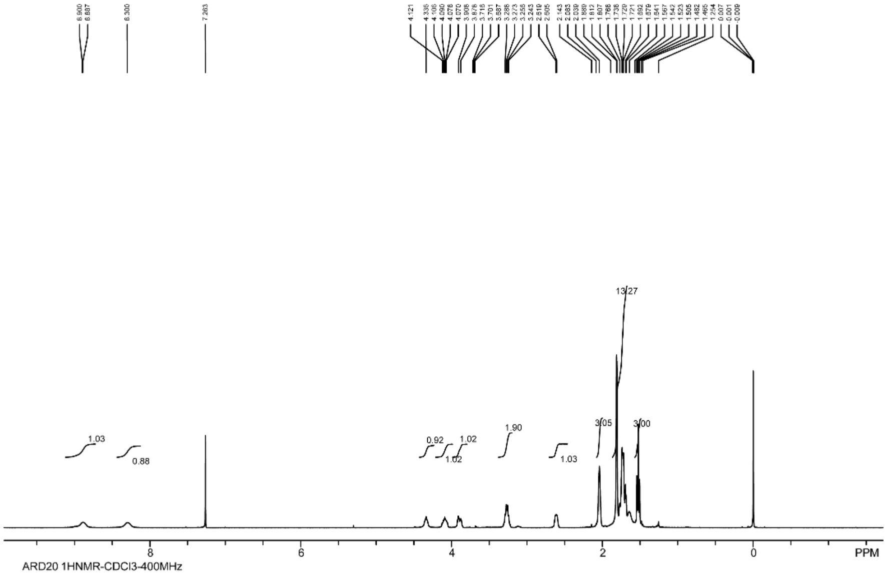

2-((3r,5r,7r)-adamantan-1-yl)propane-1,2-diamine dihydrochloride (**ARD22**)

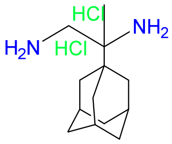

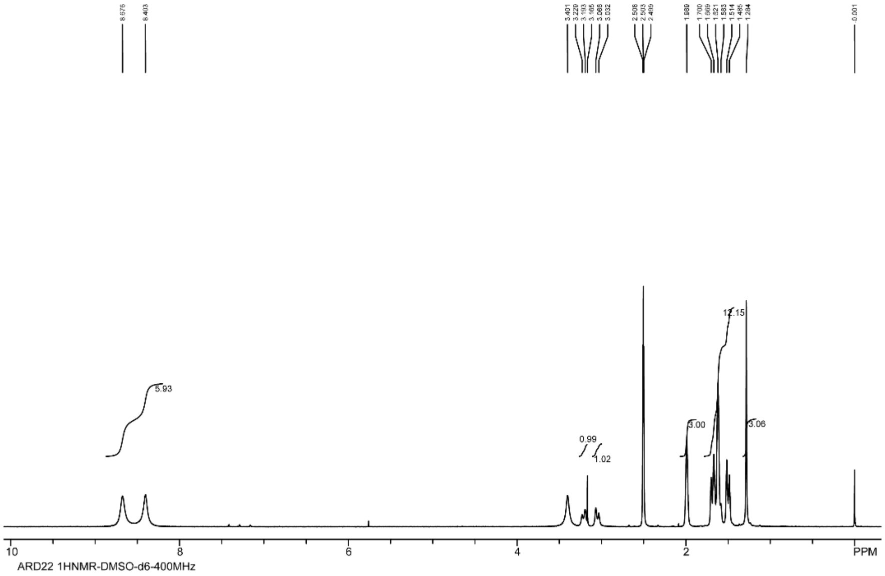

1-((3r,5r,7r)-adamantan-1-yl)-2-(1H-imidazol-1-yl)ethan-1-amine (**ARD23**)

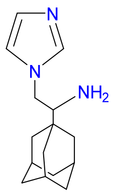

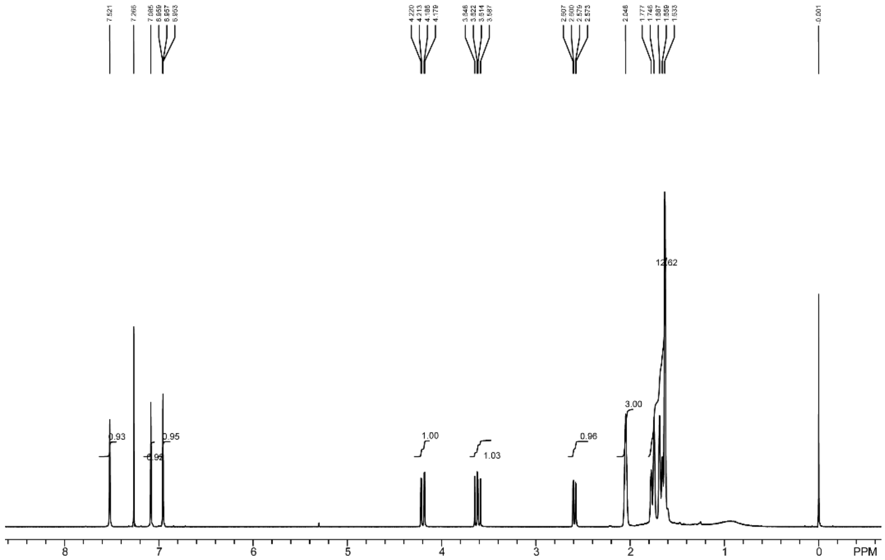

1-((3r,5r,7r)-adamantan-1-yl)-2-(1H-pyrazol-1-yl)ethan-1-amine (**ARD24**)

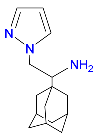

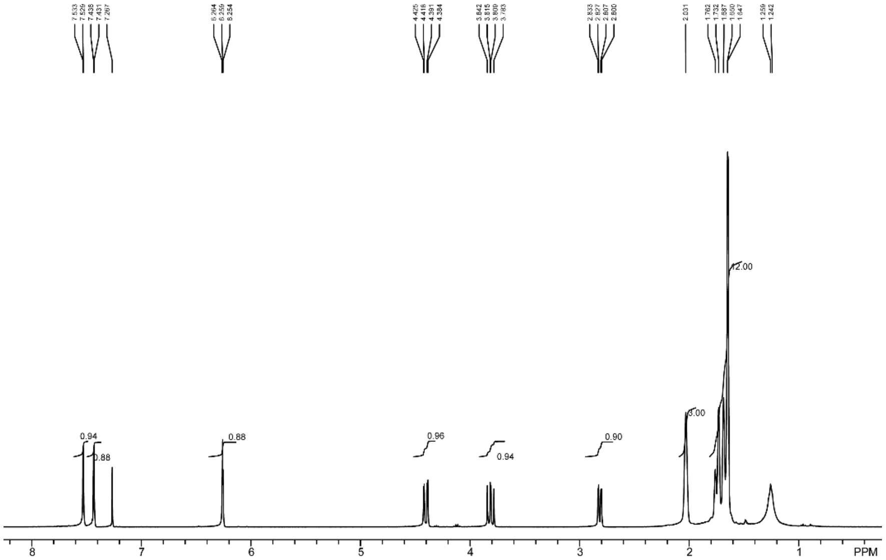

1-((3r,5r,7r)-adamantan-1-yl)-2-(1H-1,2,4-triazol-1-yl)ethan-1-amine (**ARD25**)

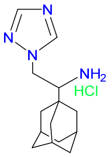

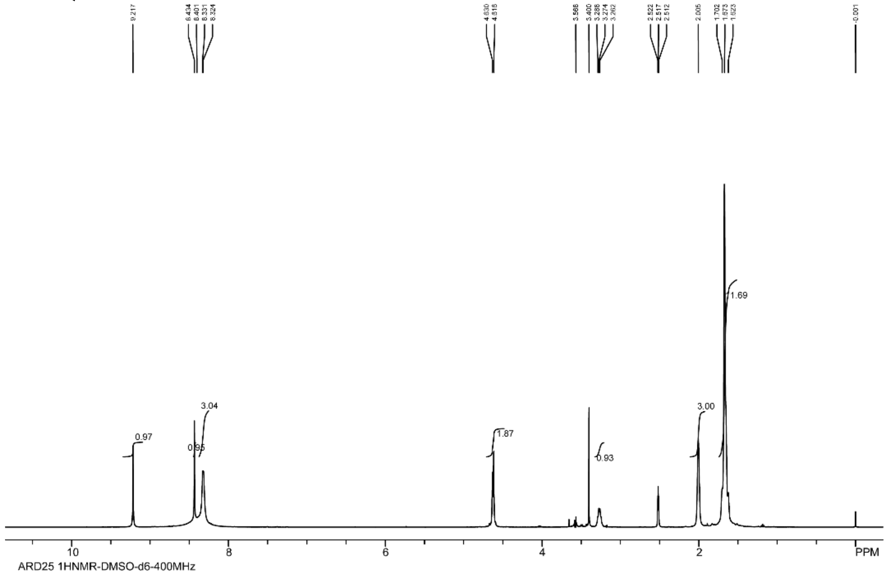

(1s,3s,5R,7S)-2-methyladamantan-1-amine (**ARD33**)

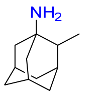

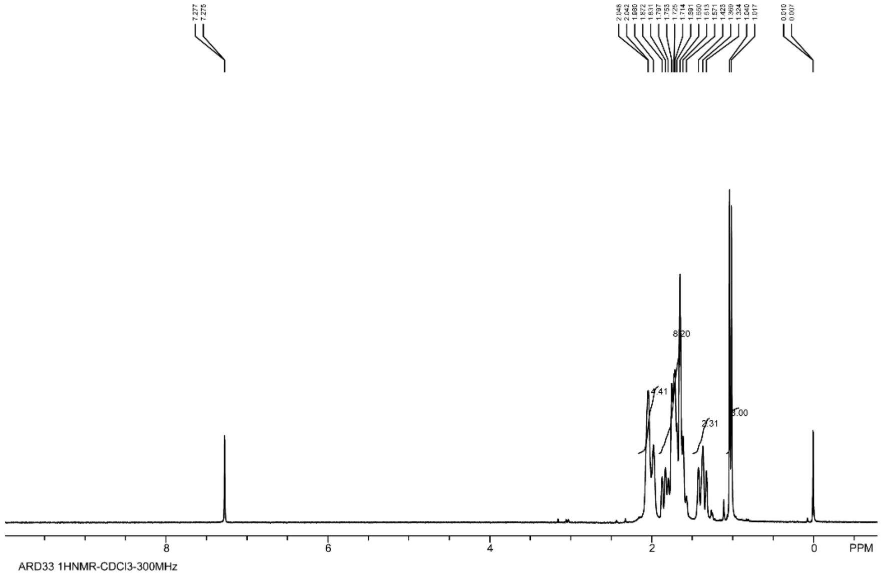

(1s,3s,5R,7S)-1-aminoadamantan-2-ol (**ARD37**)

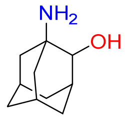

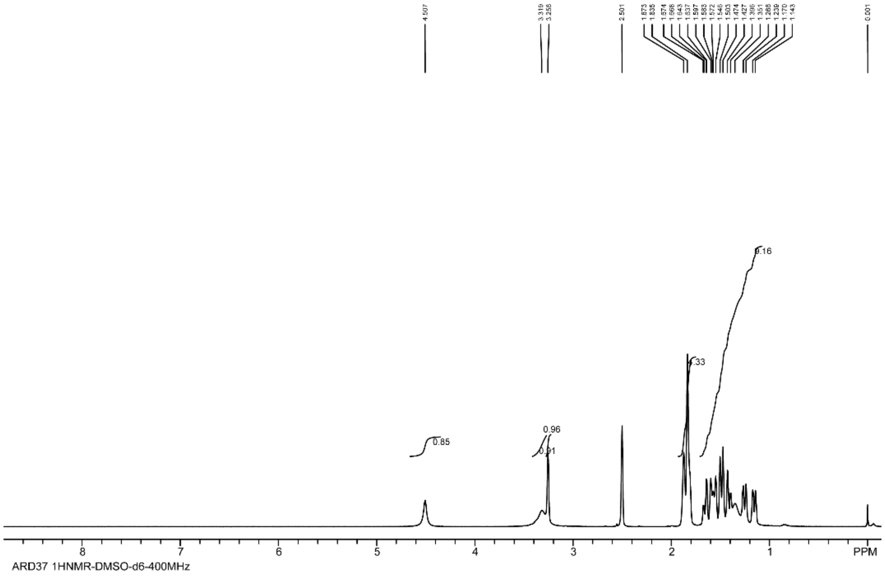

((1r,3r,5r,7r)-2-aminoadamantan-2-yl)methanol (**ARD39**)

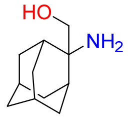

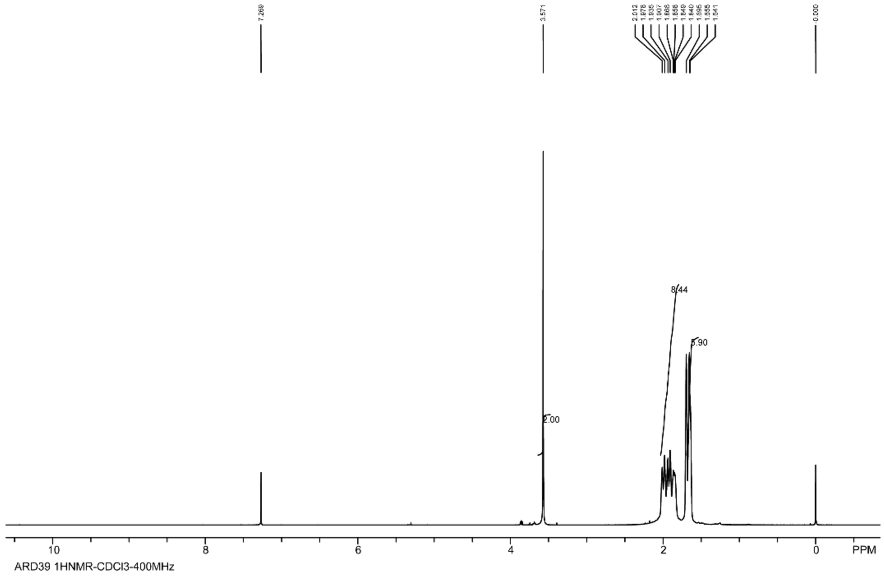

((1r,3r,5r,7r)-2-(methylamino)adamantan-2-yl)methanol (**ARD40**)

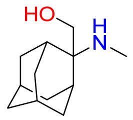

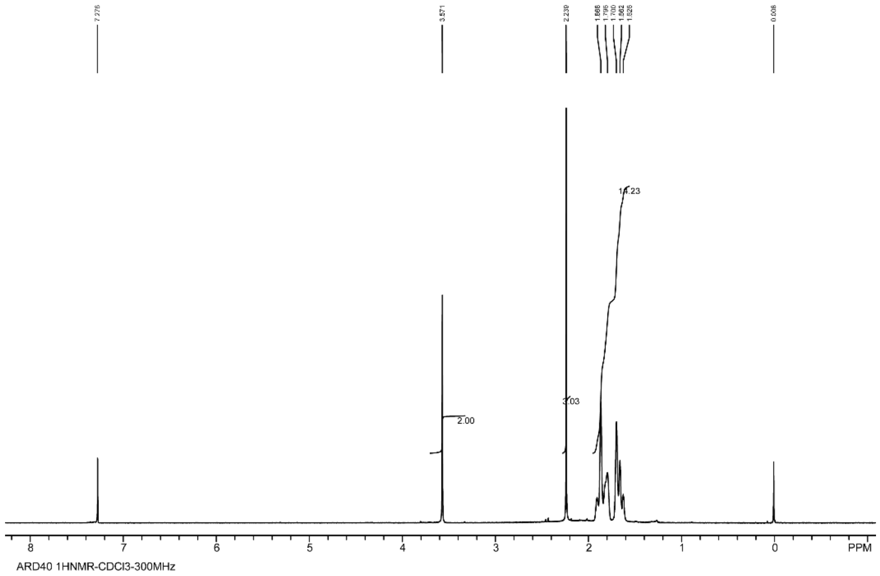

(1r,3r,5r,7r)-2-(aminomethyl)adamantan-2-amine (**ARD41**)

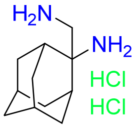

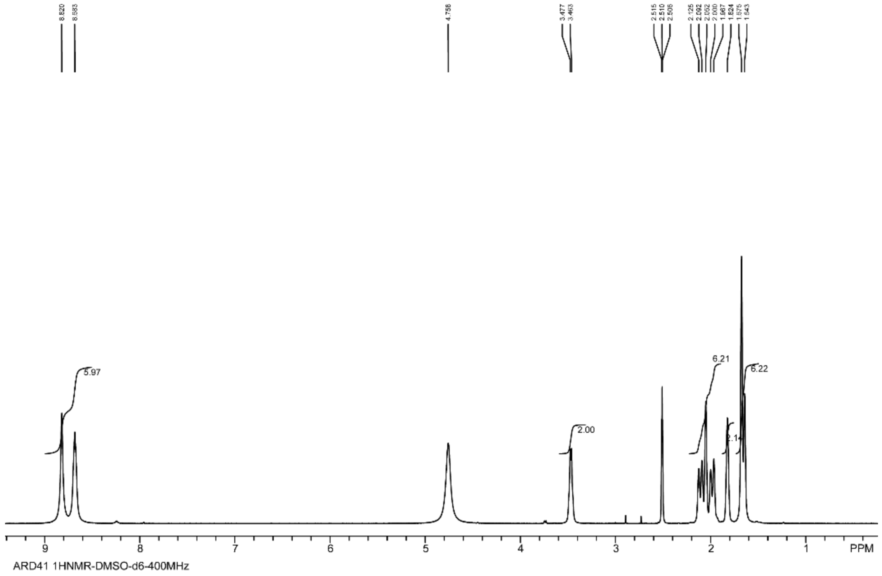

(1r,3r,5r,7r)-2-(aminomethyl)adamantan-2-ol (**ARD42**)

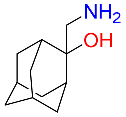

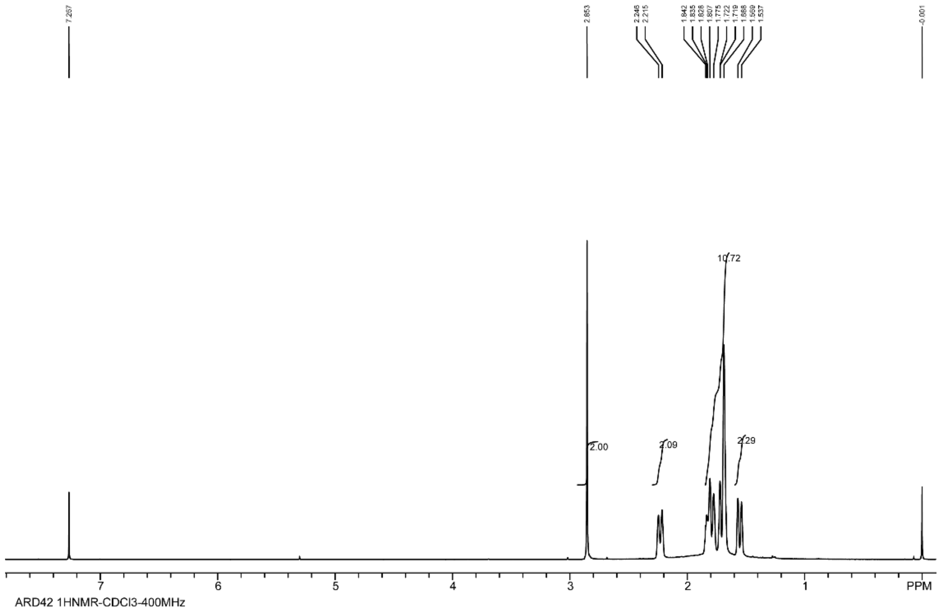

5-((3r,5r,7r)-adamantan-1-yl)-5-(hydroxymethyl)pyrrolidin-2-one (**ARD49**)

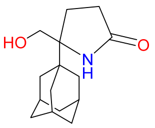

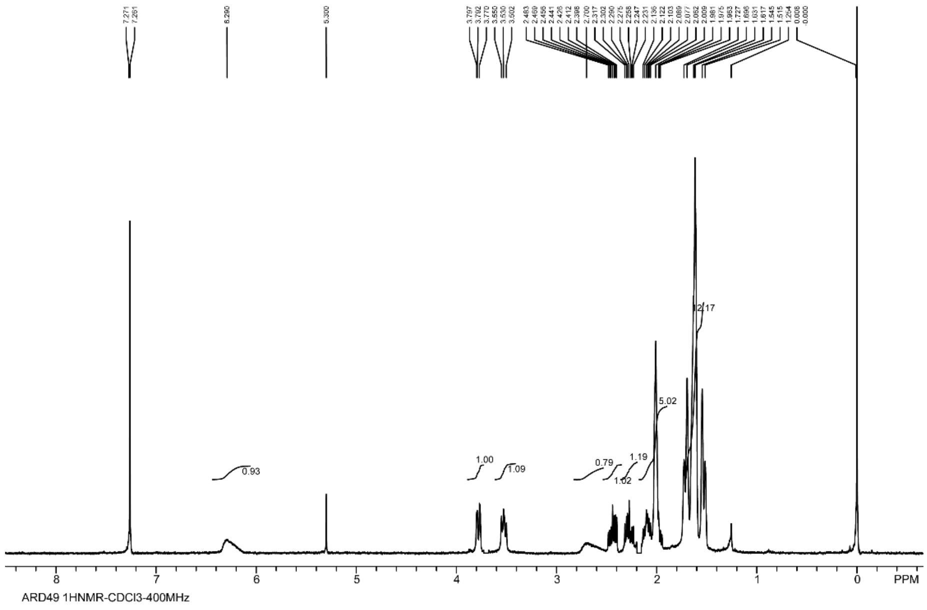

(2-((3r,5r,7r)-adamantan-1-yl)-1-methylpyrrolidin-2-yl)methanol (**ARD50**)

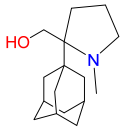

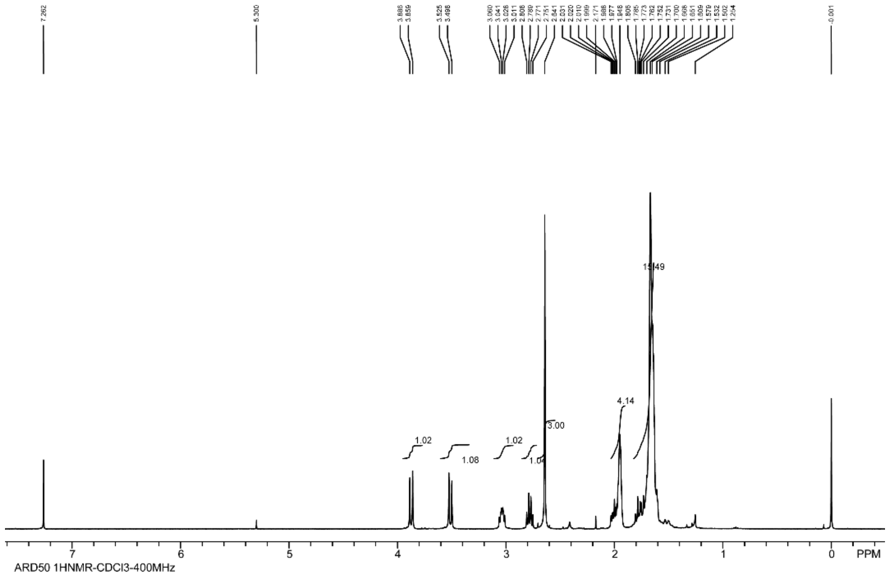

(2-((3r,5r,7r)-adamantan-1-yl)-1-ethylpyrrolidin-2-yl)methanol (**ARD51**)

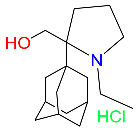

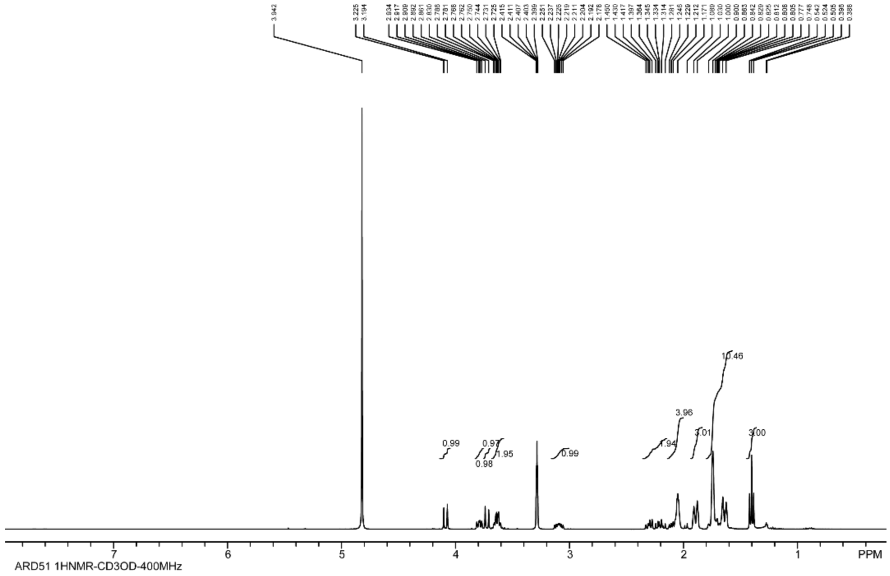

(3-((3r,5r,7r)-adamantan-1-yl)morpholin-3-yl)methanol (**ARD58**)

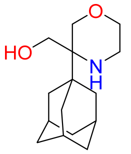

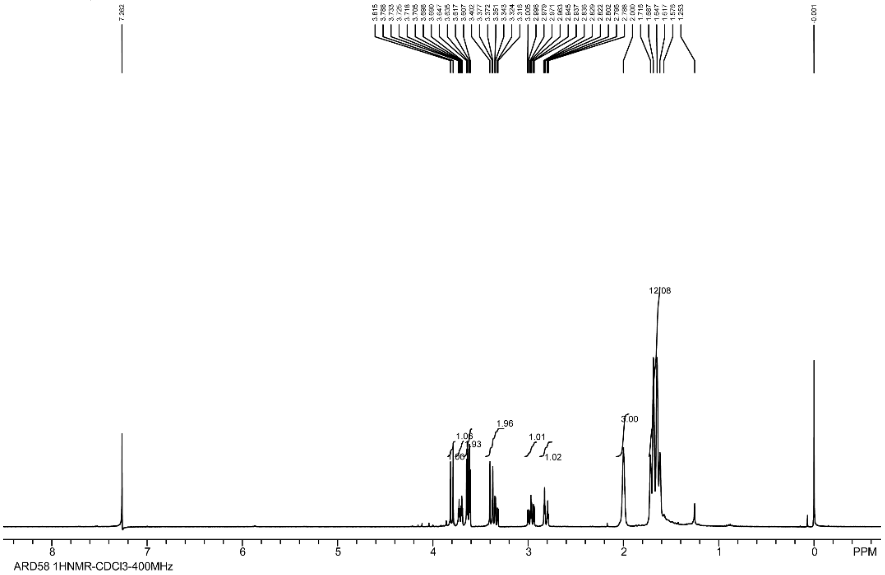

5-((3r,5r,7r)-adamantan-1-yl)-5-(hydroxymethyl)morpholin-3-one (**ARD66**)

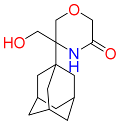

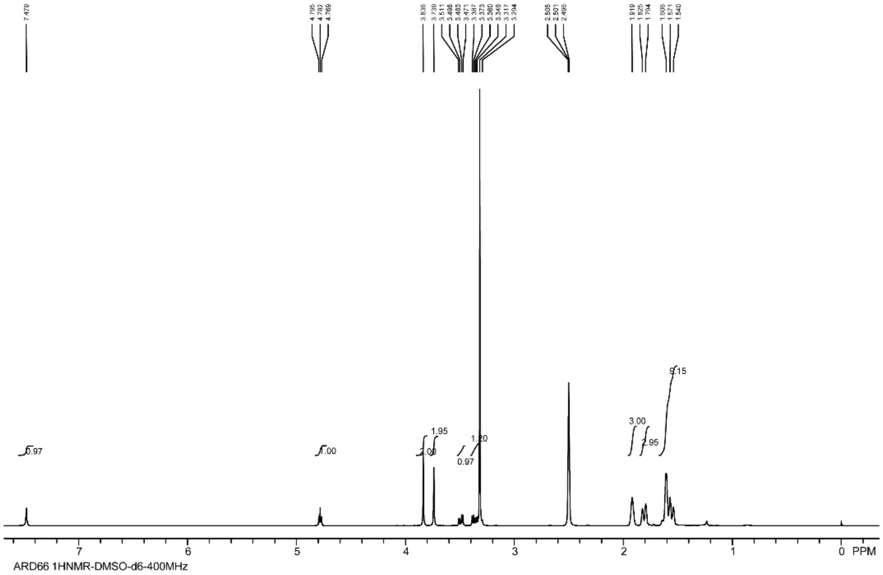

tert-butyl 2-((3r,5r,7r)-adamantan-1-yl)-2-amino-3-hydroxypropanoate (**ARD68**)

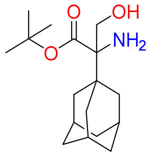

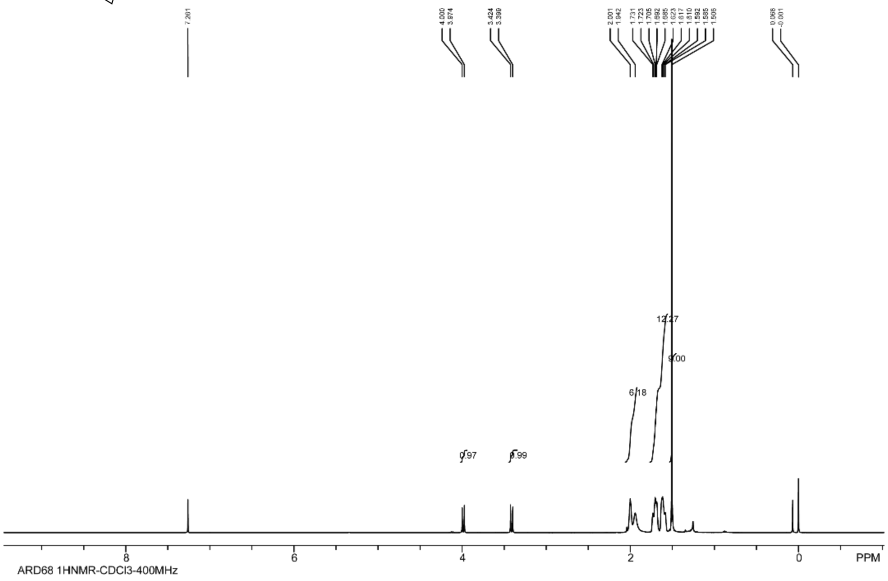

1-((3r,5r,7r)-adamantan-1-yl)-2,2-difluoroethan-1-amine (**ARD71**)

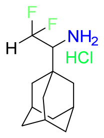

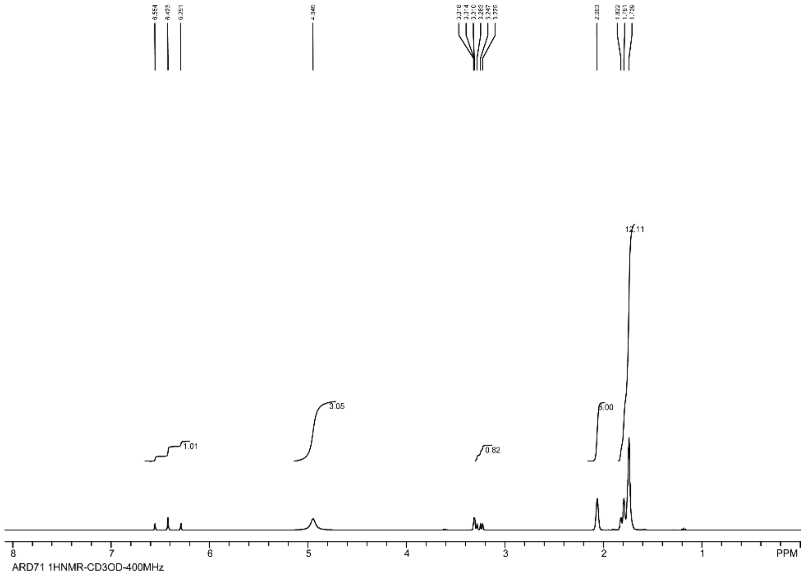

N-(2-((1R,3r)-adamantan-1-yl)-2-aminoethyl)-3-(2-(2-(3-((2-(adamantan-1-yl)-2-aminoethyl)amino)-3-oxopropoxy)ethoxy)ethoxy)propanamide (**ARD80**)

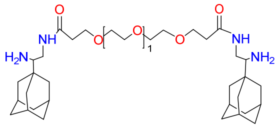

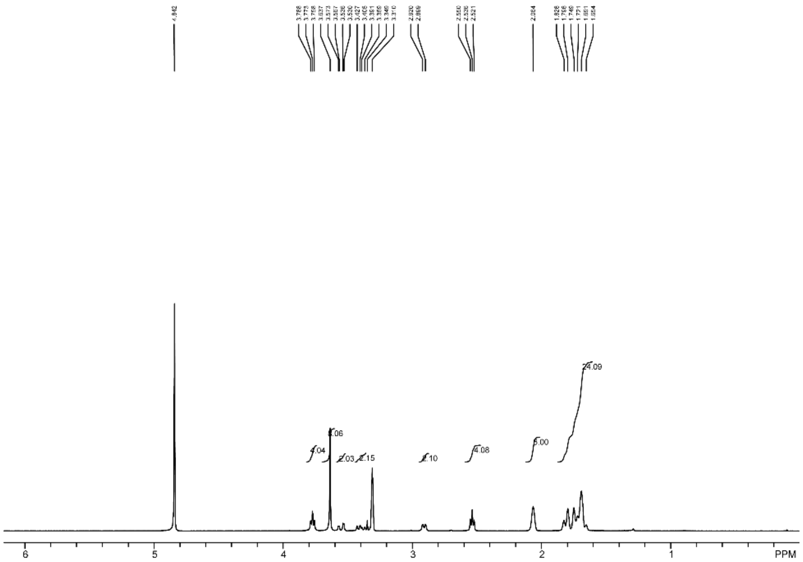

ARD80 1HNMR-CD3OD-400MHz

N1-(2-((1R,3r)-adamantan-1-yl)-2-aminoethyl)-N16-(2-(adamantan-1-yl)-2-aminoethyl)-4,7,10,13-tetraoxahexadecanediamide (**ARD81**)

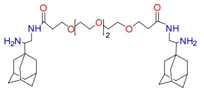

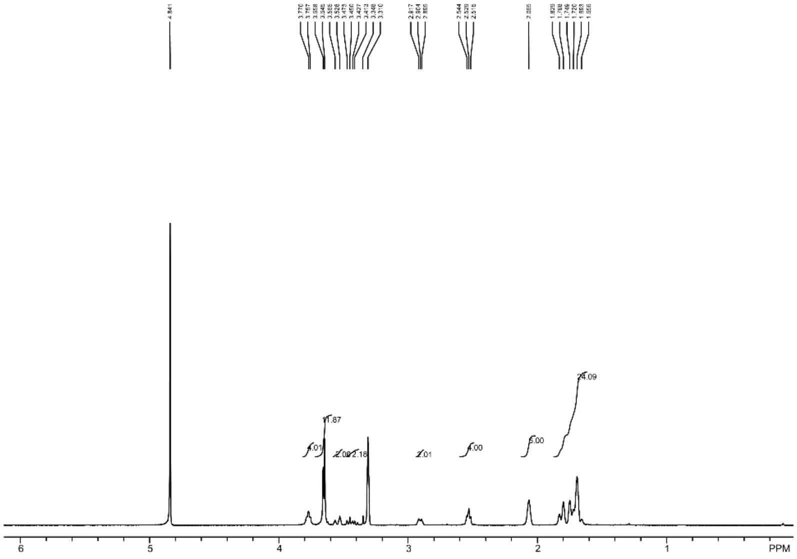

ARD81 1HNMR-CD3OD-400MHz

N1-(2-((1R,3r)-adamantan-1-yl)-2-aminoethyl)-N22-(2-(adamantan-1-yl)-2-aminoethyl)-4,7,10,13,16,19-hexaoxadocosanediamide (**ARD83**)

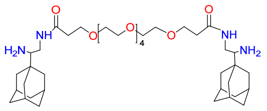

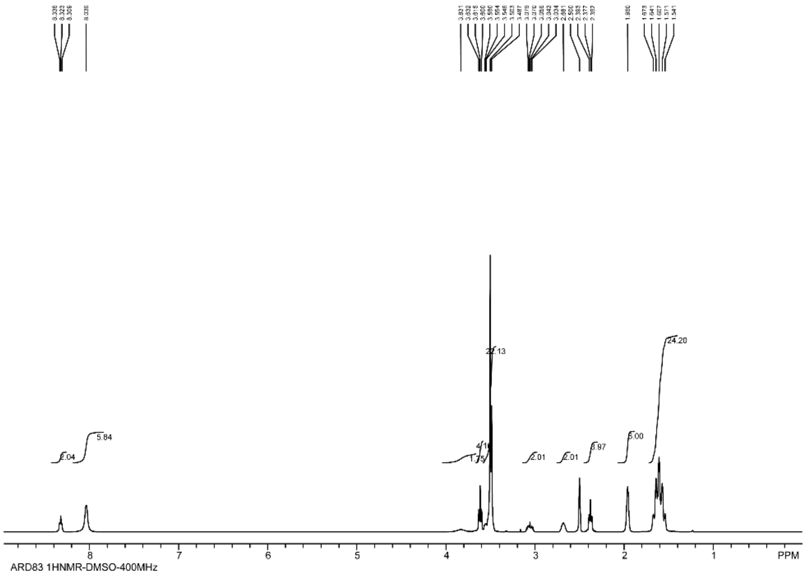

N1-(2-((1R,3r)-adamantan-1-yl)-2-aminoethyl)-N25-(2-(adamantan-1-yl)-2-aminoethyl)-4,7,10,13,16,19,22-heptaoxapentacosanediamide (**ARD84**)

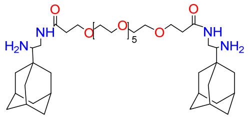

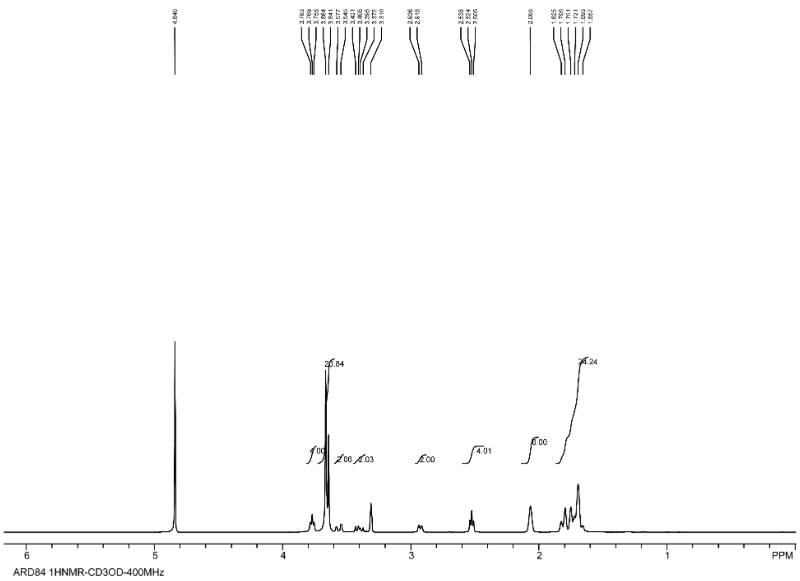

3,3'-oxybis(N-(2-(adamantan-1-yl)-2-aminoethyl)propanamide) (**ARD87**)

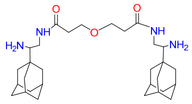

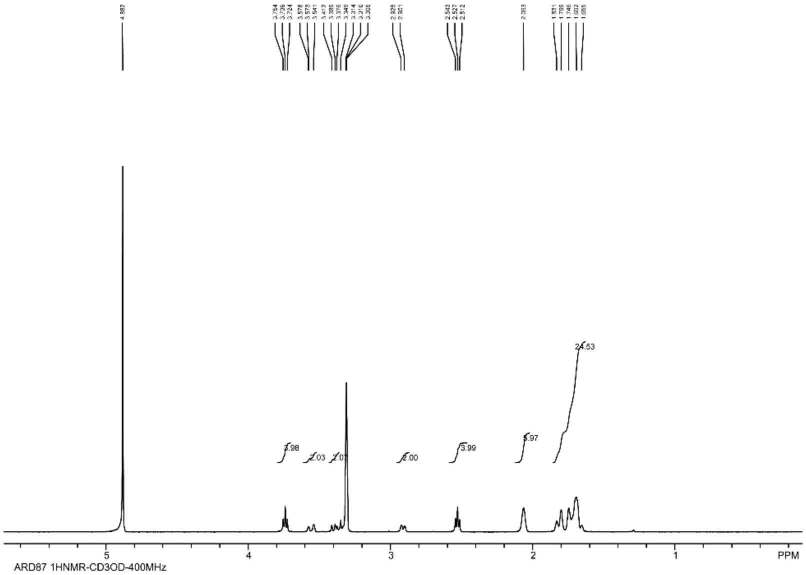

(3s,5s,7s)-N-((5-cyclopropylisoxazol-3-yl)methyl)adamantan-1-amine (**ARD88**)

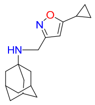

4-((((3s,5s,7s)-adamantan-1-yl)amino)methyl)benzene-1,3-diol (**ARD89**)

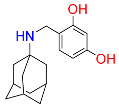

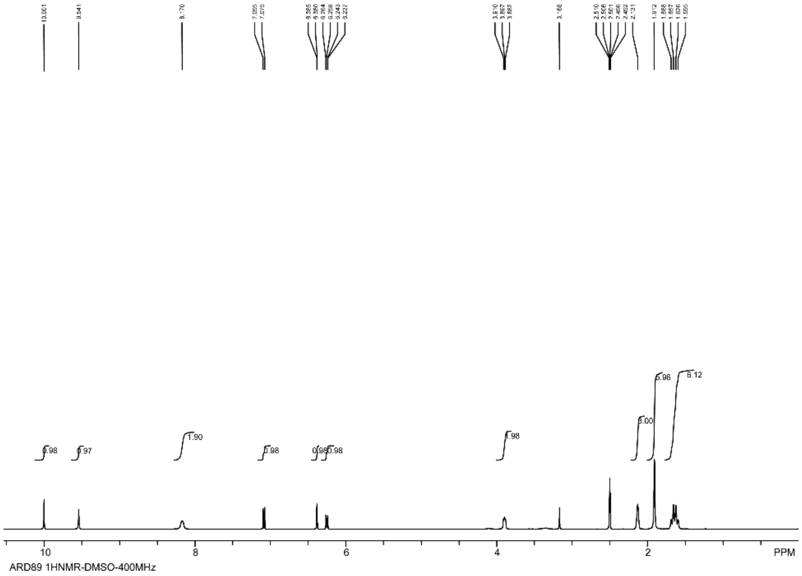

((1r,3r,5r,7r)-2-((((5-phenylisoxazol-3-yl)methyl)amino)methyl)adamantan-2-yl)methanol (**ARD98**)

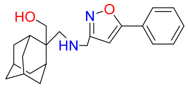

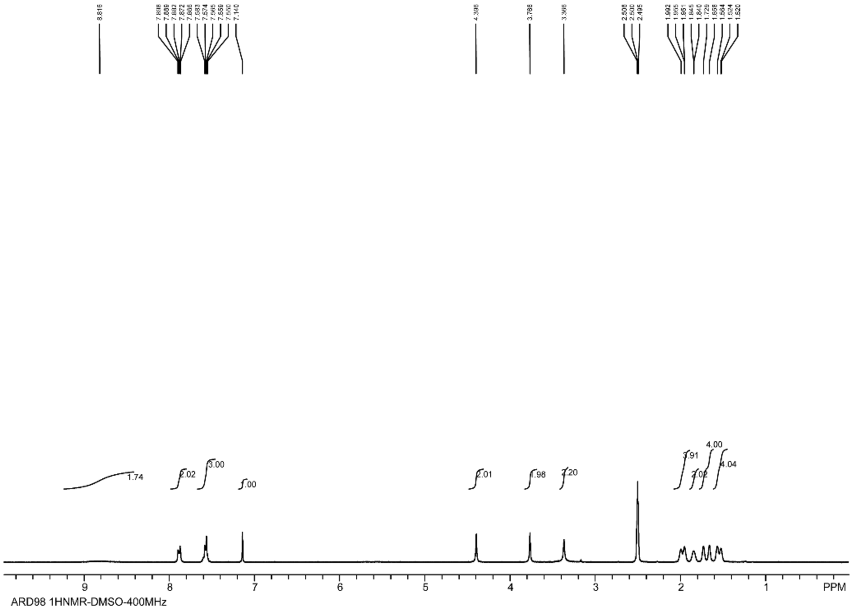

((1r,3r,5r,7r)-2-((((2-methylpyridin-4-yl)methyl)amino)methyl)adamantan-2-yl)methanol (**ARD99**)

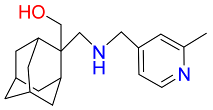

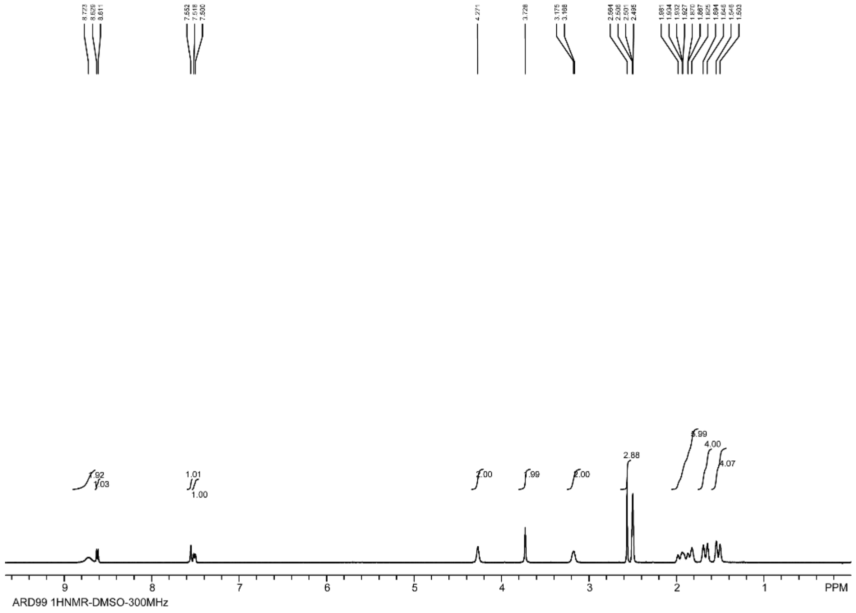

((1r,3r,5r,7r)-2-((((6-methylpyridin-3-yl)methyl)amino)methyl)adamantan-2-yl)methanol (**ARD100**)

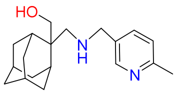

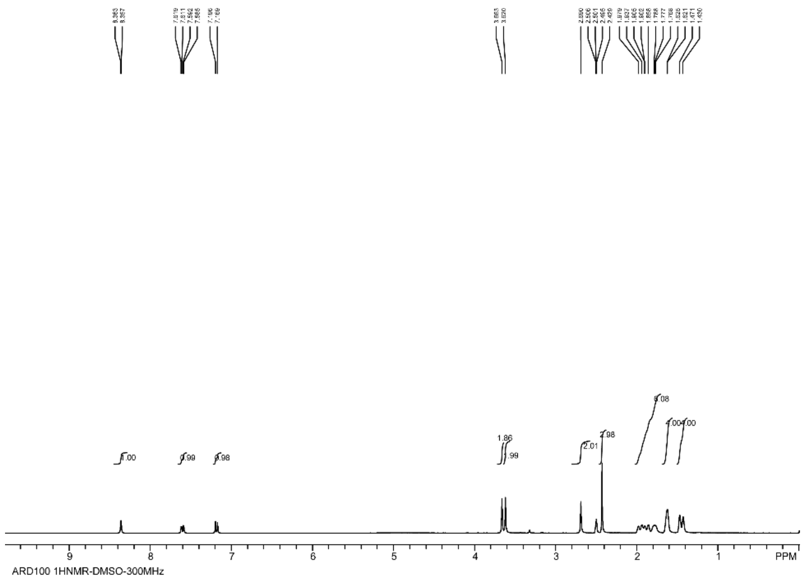

((1r,3r,5r,7r)-2-((((5-cyclopropylisoxazol-3-yl)methyl)amino)methyl)adamantan-2-yl)methanol (**ARD101**)

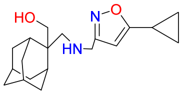

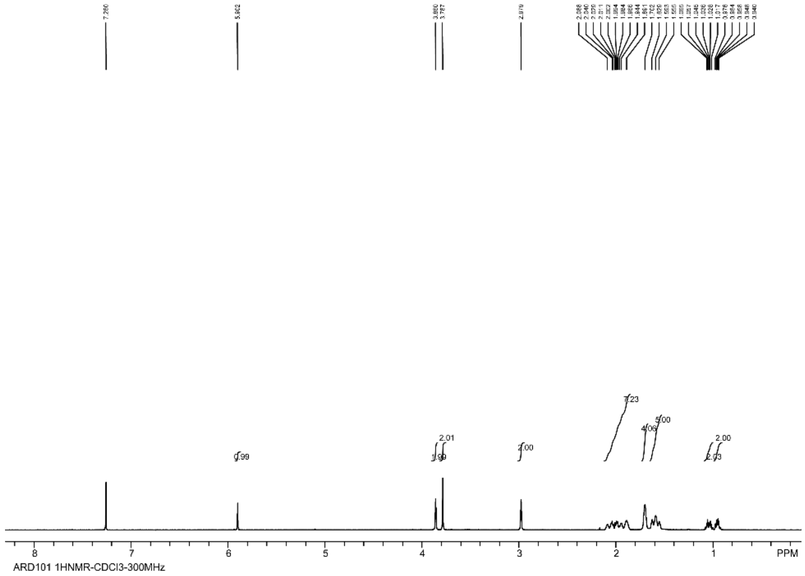

3,5-dihydroxy-N-(1-imino-2-(((1r,3r,5r,7r)-2-methyladamantan-2-yl)amino)ethyl)benzimidamide (**ARD112**)

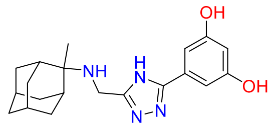

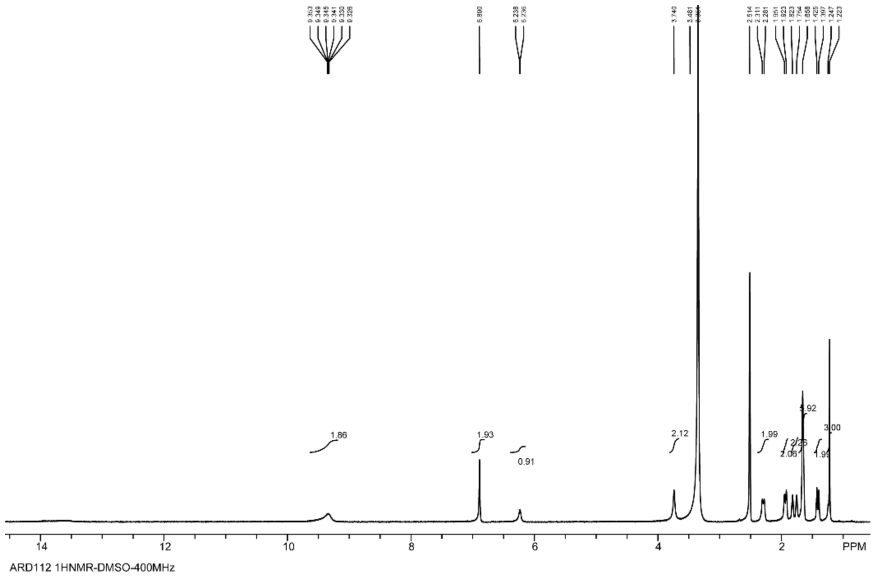

References

1. Shiryaev, V.A.; Radchenko, E.V.; Palyulin, V.A.; Zefirov, N.S.; Bormotov, N.I.; Serova, O.A.; Shishkina, L.N.; Baimuratov, M.R.; Bormasheva, K.M.; Gruzd, Y.A., et al. Molecular design, synthesis and biological evaluation of cage compound-based inhibitors of hepatitis C virus p7 ion channels. *Eur J Med Chem* **2018**, *158*, 214-235, doi:10.1016/j.ejmech.2018.08.009.
2. Mandour, Y.M.; Breitinger, U.; Ma, C.; Wang, J.; Boeckler, F.M.; Breitinger, H.G.; Zlotos, D.P. Symmetric dimeric adamantanes for exploring the structure of two viroporins: influenza virus M2 and hepatitis C virus p7. *Drug Des Devel Ther* **2018**, *12*, 1019-1031, doi:10.2147/DDDT.S157104.
